# Fungi have three tetraspanin families with distinct functions

**DOI:** 10.1186/1471-2164-9-63

**Published:** 2008-02-03

**Authors:** Karine Lambou, Didier Tharreau, Annegret Kohler, Catherine Sirven, Mélanie Marguerettaz, Crystel Barbisan, Adrienne C Sexton, Ellen M Kellner, Francis Martin, Barbara J Howlett, Marc J Orbach, Marc-Henri Lebrun

**Affiliations:** 1 UMR 5240 CNRS-UCB-INSA-Bayer CropScience, Microbiologie, Adaptation et Pathogénie, Bayer CropScience, 14-20 rue Pierre Baizet, 69263 Lyon Cedex 09, France; 2 UMR BGPI, CIRAD-INRA-SupAgro.M, TA A 54/K, Campus International de Baillarguet, 34398 Montpellier Cedex 5, France; 3 UMR INRA/Université Henri Poincaré 1136, Interactions Arbres/Micro-organismes, Centre INRA de Nancy, 54280 Champenoux, France; 4 Bayer CropScience AG, Alfred Nobel Strasse 50, 40789 Monheim am Rhein, Germany; 5 School of Botany, The University of Melbourne, Parkville VIC 3010, Australia; 6 University of Arizona, 303 Forbes Building, P.O. Box 210036, Tucson, AZ 85721-0036, USA

## Abstract

**Background:**

Tetraspanins are small membrane proteins that belong to a superfamily encompassing 33 members in human and mouse. These proteins act as organizers of membrane-signalling complexes. So far only two tetraspanin families have been identified in fungi. These are Pls1, which is required for pathogenicity of the plant pathogenic ascomycetes, *Magnaporthe grisea*, *Botrytis cinerea *and *Colletotrichum lindemuthianum*, and Tsp2, whose function is unknown. In this report, we describe a third family of tetraspanins (Tsp3) and a new family of tetraspanin-like proteins (Tpl1) in fungi. We also describe expression of some of these genes in *M. grisea *and a basidiomycete, *Laccaria bicolor*, and also their functional analysis in *M. grisea*.

**Results:**

The exhaustive search for tetraspanins in fungal genomes reveals that higher fungi (basidiomycetes and ascomycetes) contain three families of tetraspanins (Pls1, Tsp2 and Tsp3) with different distribution amongst phyla. Pls1 is found in ascomycetes and basidiomycetes, whereas Tsp2 is restricted to basidiomycetes and Tsp3 to ascomycetes. A unique copy of each of *PLS1 *and *TSP3 *was found in ascomycetes in contrast to *TSP2*, which has several paralogs in the basidiomycetes, *Coprinus cinereus *and *Laccaria bicolor*. A tetraspanin-like family (Tpl1) was also identified in ascomycetes. Transcriptional analyses in various tissues of *L. bicolor *and *M. grisea *showed that *PLS1 *and *TSP2 *are expressed in all tissues in *L. bicolor *and that *TSP3 *and *TPL1 *are overexpressed in the sexual fruiting bodies (perithecia) and mycelia of *M. grisea*, suggesting that these genes are not pseudogenes. Phenotypic analysis of gene replacementmutants *Δtsp3 *and *Δtpl1 *of *M. grisea *revealed a reduction of the pathogenicity only on rice, in contrast to *Δpls1 *mutants, which are completely non-pathogenic on barley and rice.

**Conclusion:**

A new tetraspanin family (Tsp3) and a tetraspanin-like protein family (Tpl1) have been identified in fungi. Functional analysis by gene replacement showed that these proteins, as well as Pls1, are involved in the infection process of the plant pathogenic fungus *M. grisea*. The next challenge will be to decipher the role(s) of tetraspanins in a range of symbiotic, saprophytic and human pathogenic fungi.

## Background

Tetraspanins are a superfamily of small integral membrane proteins that were first identified in mammals as cell-specific antigens [1] and, since then, in fishes, insects, worms, sponges [2] and fungi [2,3]. They are not found in plants, although tetraspanin-like proteins were identified in *Arabidopsis thaliana *[4,5]. To date, at least 33 distinct tetraspanins have been identified in humans, 37 in *Drosophila melanogaster *and 20 in *Caenorhabditis elegans *[2]. Animal tetraspanins interact tightly and specifically with other membrane proteins and have been proposed to act as molecular facilitators of membrane protein complexes [1,6]. Animal tetraspanins are also able to interact with other tetraspanins to form a scaffold that promotes the recruitment of tetraspanin-associated proteins, hence creating a web [7]. As a consequence of this complexity, tetraspanin webs from different cell types differ markedly in their protein composition and probably also in their cellular function. This may explain the diversity of phenotypes observed among tetraspanin knockout mutants in animals. Indeed, tetraspanin null mutants are altered in diverse unrelated processes such as sperm-egg fusion, susceptibility to parasites or viruses, neuronal or lymphocyte cell-cell interactions, cell adhesion, motility and polarity and protein trafficking [1,6,8].

**Figure 1:**
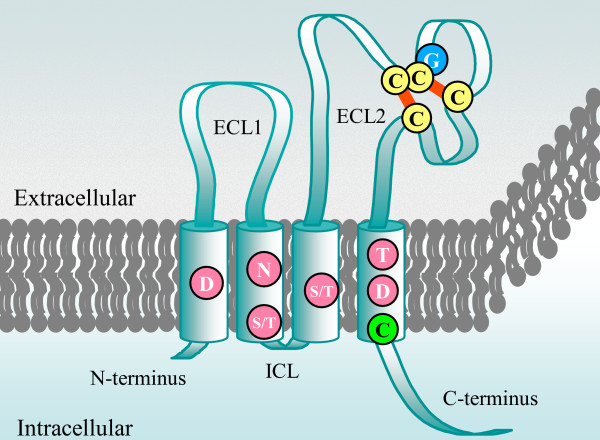
**Schematic structure of Pls1 tetraspanins**. Tetraspanins contain four transmembrane domains (TM1 to TM4) with conserved polar/charged residues (pink circles), a small extracellular loop (ECL1), a small intracellular loop (ICL) and a large extracellular loop (ECL2). ECL2 contains a CCG motif and further two conserved cysteine residues (yellow circles). The N-terminal and C-terminal tails are intracellular. These cysteine residues allow formation of two disulphide bridges (red lines), crucial for the folding of ECL2. One putative palmitoylation site is located proximal to TM4 (green circle).

Although tetraspanins display limited amino acid sequence similarity, they share conserved structural hallmarks consisting of four transmembrane domains (TM1 to TM4), a small extracellular loop (ECL1), an intracellular loop (ICL) and a large extracellular loop (ECL2) containing a characteristic cysteine-based pattern [9,10]. In fungi, the first tetraspanin identified was in the plant pathogenic fungus *M. grisea *and was named Pls1 ([3]; Figure 1). Since then, genes homologous to *PLS1 *have been identified in other ascomycetes [11] and basidiomycetes [2]. The functional role of Pls1 in the fungal cell has been investigated in three plant pathogenic ascomycetes [12], namely *M. grisea *(MgPls1, [3]), *Botrytis cinerea *(BcPls1, [13]) and *Colletotrichum lindemuthianum *(ClPls1, [14]). These fungi are responsible for major diseases of agriculturally important plants, *M. grisea *causing the most devastating fungal disease of rice (blast) worldwide, *B. cinerea *being a necrotrophic pathogen affecting more than 200 different host plants and *C. lindemuthianum *causing anthracnose diseases on a wide range of crops and ornamental plants. During the infection process, these fungi differentiate a specialized cell called the appressorium that is able to perforate the plant cuticle and cell wall, allowing the fungus to penetrate into host tissues. The *ΔMgpls1*, *ΔBcpls1 *and *ΔClpls1 *null mutants were all non-pathogenic on intact host plant tissues. Although the mutants could differentiate appressoria, these infection structures were unable to direct the penetration of the fungus into host plant tissues [3,13,14]. These results demonstrate that Pls1 tetraspanins are involved in a function conserved among fungi that is essential for appressorium-mediated penetration.

Previous studies suggested that fungal genomes possess a single tetraspanin gene [11]. However, analysis of recently sequenced fungal genomes revealed that besides Pls1 orthologs, basidiomycetes have other tetraspanins called Tsp2 [2]. This prompted us to systematically search for novel tetraspanins in fungal genomes and EST databases to better characterize this protein family in fungi. Using low stringency BlastP and HMM (Hidden Markov Model) searches using the PFAM tetraspanin profile (PF00335), we identified a novel tetraspanin (Tsp3) and a tetraspanin-like protein (Tpl1) in *M. grisea *and their orthologs in other ascomycetes. Furthermore, genes from the *TSP2 *family were identified in newly available basidiomycete genomes and they displayed a complex evolutionary history with numerous paralogs. The expression profiles of *PLS1, TSP2, TSP3 *and *TPL1 *were determined by quantitative RT-PCR in *M. grisea *or by microarray analysisin the symbiotic *L. bicolor*. Expression of these genes was also estimated *in silico *by compiling their occurrence in EST or cDNA libraries. Finally, a functional study of *TSP3 *and *TPL1 *was performed in *M. grisea*, whereby deletion mutants were constructed by targeted gene replacement and compared to the existing *Δpls1 *mutant. In marked contrast to this latter mutant, which was fully non-pathogenic on barley and rice, the *Δtsp3 *and *Δtpl1 *mutants were reduced in pathogenicity only on rice. Thus deletion of these genes has a host-specific effect on the pathogenicity of *M. grisea *which is different from that of the *PLS1 *deletion.

## Results

### Pls1 tetraspanins are widespread in ascomycetes and basidiomycetes

Proteins homologous to Pls1 [see Additional file 1] and [Additional file 2] were identified using Blastp in all available sequenced genomes of ascomycetes (*Sclerotinia sclerotiorum*, *Leptosphaeria maculans*, *Podospora anserina*, *Chaetomium globosum*, *Trichoderma reesei, Fusarium verticilloides, Nectria haematococca, Stagonospora nodorum*, *Coccidioides posadasii) *and basidiomycetes (*Laccaria bicolor*), except *Ustilago maydis, Cryptoccocus neoformans, Aspergillus *species, *Mycosphaerella graminicola*, and all *Saccharomycotina *species (hemi-ascomycetes). Another strategy based on the use of the tetraspanin HMM profile (PF00335) revealed a single Pls1 tetraspanin in the same fungal genomes. *SsPLS1*, *LmPLS1, PaPLS1, GzPLS1, CpPLS1 *cDNAs were obtained either from cDNA libraries or reconstructed from ESTs and used to define introns and start and stop codons in the corresponding genes.

**Figure 2:**
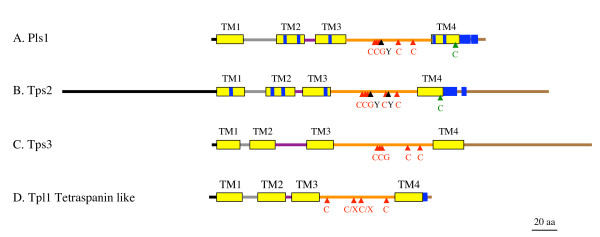
**Structural comparison of fungal tetraspanins (Pls1, Tsp2, Tsp3) and tetraspanin like proteins (Tpl1)**. The Pls1, Tsp2 and Tsp3 tetraspanin families display the typical structure of tetraspanin: four transmembrane domains (TM1, TM2, TM3, TM4, yellow boxes) and a small extracellular loop (ECL1, gray line), a small intracellular loop (ICL, purple line), a large extracellular loop (ECL2, orange line), intracellular N-terminal (black line) and C-terminal (brown line) tails. ECL2 contains a CCG motif and further two conserved cysteine residues (red triangles). A. The Pls1 tetraspanin family contains conserved polar/charged residues in TM1, TM2, TM3 and TM4 (blue lines) and one putative palmitoylation site is proximal to TM4 (green triangle). B. The Tsp2 tetraspanin family displays large intracellular N-terminal and C-terminal tails. One putative conserved palmitoylation site is located at the end of TM4 (green triangle). C. The Tsp3 tetraspanin family has a short ECL1 and a large C-terminal tail. D. The Tpl1 tetraspanin-like proteins lack the typical cysteine-based pattern in their ECL2 (CCG motif and further two conserved cysteine residues). Nevertheless, their ECL2 regions contain two conserved cysteine residues close to TM3 and TM4.

The sequence conservation between the different Pls1 tetraspanins is essentially in the TM domains, some regions of the ECL2 including its cysteine-based pattern (CCGY-x(13)-C-x(11/19)-C-x(14)-TM4) and the C-terminal tail (Figure 2, [Additional file 1] and [Additional file 2]). The ECL1 sequences are more variable, as those in basidiomycete Pls1 proteins (17 aa) are shorter than in ascomycetes (26 aa). Similarly, the conserved motif in ICL from Pls1 proteins of basidiomycetes (QRNHVTLGLV) is very different from that found in Pls1 from ascomycetes (RGWLK). Three TM domains contain charged/polar amino acids (D in TM1, S/T in TM2, T in TM4) conserved among all Pls1 proteins. Some Pls1 proteins from ascomycetes have additional charged/polar amino acids (N in TM2, S/T in TM3 and D in TM4; see Figure 1 and [Additional file 1]). In animal tetraspanins, these conserved polar/charged amino acids are thought to stabilize interactions between transmembrane domains [10]. A putative palmitoylation site (cysteine residue) is located at the end of TM4 in most Pls1 tetraspanins, except those from the Leotiomycetes, *Botrytis cinerea *and *Sclerotinia sclerotiorum *(Figures 1 and 2, [Additional file 1]). In animals, the palmitoylation of several conserved juxtamembrane cysteine residues influences the formation of tetraspanin-enriched membrane microdomains [15-17]. A variable region is located in the ECL2 loop of fungal Pls1 tetraspanins (Figures 1 and 2, [Additional file 1]), between the last two cysteine residues whereas, it is located between the second and the last cysteine residues of the ECL2 in animal tetraspanins. Interestingly, in animals this variable region contains sites involved in specific tetraspanin protein-protein interactions [10]. The cysteine-based pattern of fungal Pls1 tetraspanins (CCGY-x(13)-C-x(11/19)-C-x(14)-TM4) contains a conserved tyrosine residue located after the CCG motif that is not in the cysteine-based pattern of animal tetraspanins (Figures 1 and 2, [Additional file 1] and [Additional file 2]). The cysteine-based pattern of ascomycete Pls1 EC2 (CCGY-x(13)-C-x(10)-GC-x(14)-TM4) is slightly different from that found in Pls1 from basidiomycetes (CCGY-x(13)-C-x(19)-C-x(14)-TM4, [Additional file 1] and [Additional file 2]) suggesting their ECL2 tertiary structure slightly differs from those of ascomycetes. The amino acid sequences of the C-terminal tail of fungal Pls1 tetraspanins are strongly conserved and are rich in charged/polar amino acids (average 70%). Indeed, these C-terminal tails display a conserved RxExERF/YxxIDxK motif found in Pls1 from ascomycetes as well as from basidiomycetes [see Additional file 1]. This conservation suggests that the C-terminal tail of fungal Pls1 tetraspanins plays a functional role as does that of animal tetraspanins [18,19].

### Tsp2 tetraspanins are restricted to basidiomycetes

Prior to this study, the first set of Tsp2 tetraspanins was identified in basidiomycetes such as *Coprinus cinereus *(CcTsp2A, CcTsp2B, CcTsp2C), *Phanerochaete chrysosporium *(PcTsp2) and *Cryptococcus neoformans *(CnTsp2, [2]). Tsp2 proteins display a long N-terminal tail (83 to 200 aa) and a long C-terminal cytoplasmic tail (66 to 101 aa). These domain lengths are characteristic of the Tsp2 family as Pls1 tetraspanins contain only short N-terminal and C-terminal tails (3–5 and 17–22 aa, respectively, Figure 2 and [Additional file 3]). Furthermore, the C-terminal tails of Tsp2 proteins are not as rich in charged amino acids (30%) as those of Pls1 proteins (60–75%). We identified four homologues of *TSP2 *(*LbTSP2A*, *LbTSP2B*, *LbTS2C *and *LbTSP2D) *in the basidiomycete *L. bicolor*. The correspondingcDNAs reconstructed from ESTs were used to define introns and start/stop codons in the corresponding genes. This family displays all the structural hallmarks of tetraspanin secondary structure with four transmembrane domains, a small extracellular loop (ECL1, 19 aa), a small intracellular loop (ICL, 4 aa), a large extracellular loop (ECL2, 72 aa) with a typical cysteine-based pattern (CCGY/F-x(12)-CY/F-x(6)-GCK-x(13)-TM4) and a conserved C-terminal cytoplasmic tail (Figure 2, [Additional file 2] and [Additional file 3]). Three TM domains contain one or two conserved charged/polar amino acids (N and Y in TM1, S and T in TM2 and Y in TM3) as observed for animal and fungal Pls1 tetraspanins [10,11]. One putative conserved palmitoylation site (a cysteine residue at the junction between a transmembrane domain and an intracellular domain) is located at the end of TM4, proximal to the inner side of the membrane according to the predicted fungal tetraspanin topology. Additionally, LbTsp2A and PcTsp2 display another putative palmitoylation site (cysteine residue) at the start of TM1, proximal to the inner side of the membrane. We were not able to identify proteins orthologous to Tsp2 in ascomycetes using blast analysis or the tetraspanin HMM profile.

### Tsp3 is a novel tetraspanin family specific to ascomycetes

**Figure 3:**
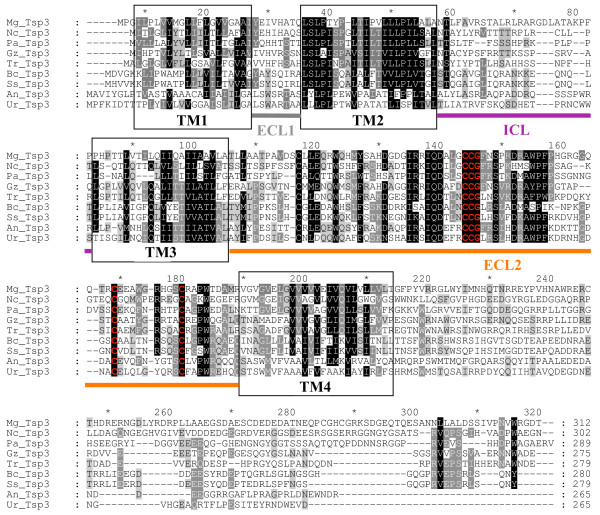
**Alignment of Tsp3 tetraspanins**. Sequences were aligned using ClustalX 1.8. Conserved amino acids are indicated in black (>80%), dark gray (>60%) and light gray (>40%). The transmembrane domains (TM) are circled in black, the small extracellular loop (ECL1), the small intracellular loop (ICL), the large extracellular loop (ECL2) are shown in gray, purple and orange lines, respectively. ECL2 contains a CCG motif and further two conserved cysteine residues (red). These cysteine residues allow formation of two disulphide bridges crucial for the folding of ECL2. Mg: *Magnaporthe grisea*, Pa: *Podospora anserina*, Nc: *Neurospora crassa*, Bc: *Botrytis cinerea*, Ss: *Sclerotinia sclerotiorum*, Tr: *Trichoderma reesei*, Gz: *Gibberella zea*, An: *Aspergillus niger*, Ur:*Uncinocarpus reesii*. For a tetraspanin structural model, see Figure 1.

We identified a novel tetraspanin in *M. grisea *which we named MgTsp3, using the tetraspanin HMM profile (PF00335). MgTsp3 is a modified version of MGG_13913.5 and annotation errors were corrected using the *TSP3 *cDNA sequence. This novel tetraspanin displays the structural hallmarks of tetraspanins including a characteristic cysteine-based pattern (CCG-x(18/22)-C-x(9)-C-x(11)-TM4) and a C-terminal tail containing a high proportion of polar/charged amino acids (70%; Figures 2 and 3). However, it differs from Pls1 of ascomycetes in the following features: a lack of charged/polar amino acid in TM domains, a smaller ECL1 (8 aa compared to 26 aa), a longer ICL (22–25 aa compared to 8 aa) and a longer C-terminal tail (70–110 aa compared to 17–22 aa) similar in size to that of Tsp2 proteins from basidiomycetes (66 to 101 aa, Figure 2).

Proteins homologous to Tsp3 were detected only in ascomycetes, including *P. anserina*, *T. reesei, N. crassa, C. globosum, G. zeae, S. nodorum*, *B. cinerea, S. sclerotiorum *and *Uncinocarpus reesii *(Figure 3). A Tsp3 homologue was identified in *Aspergillus niger*, this being the first report of an *Aspergillus *tetraspanin (Figure 3). In marked contrast, the Tsp3 protein is absent from basidiomycete genomes. The amplification of *MgTSP3 *cDNA obtained by RT-PCR allowed the identification of three introns at positions 37, 66 and 595 bp. Other *TSP3 *genes were aligned to ESTs when available [see Additional file 2], allowing prediction of their exon positions and their corresponding proteins in *N. crassa *and *S. sclerotiorum*. The number of introns varies between one and four in the Sordariaceae, with a conserved intron at position 31 bp from the start codon [see Additional file 2]. *TSP3 *genes from Leotiomycetes (*Botrytis cinerea *and *S. sclerotiorum*) have four introns at conserved positions [see Additional file 2]. *SnTsp3 *and *TrTsp3 *did notdisplay four transmembrane domains; however, this is probably due to a sequence gap and an incorrect intron annotation, respectively. For this reason, these genes were not included in the comparative analysis. Overall Tsp3 sequences are not as conserved as Pls1 and Tsp2 proteins from the same range of species (Figures 3). In particular, ICL and C-terminal tail are not conserved, unlike that in Pls1 proteins (Figures 3).

### Tpl1 is a tetraspanin-like protein restricted to ascomycetes

**Figure 4:**
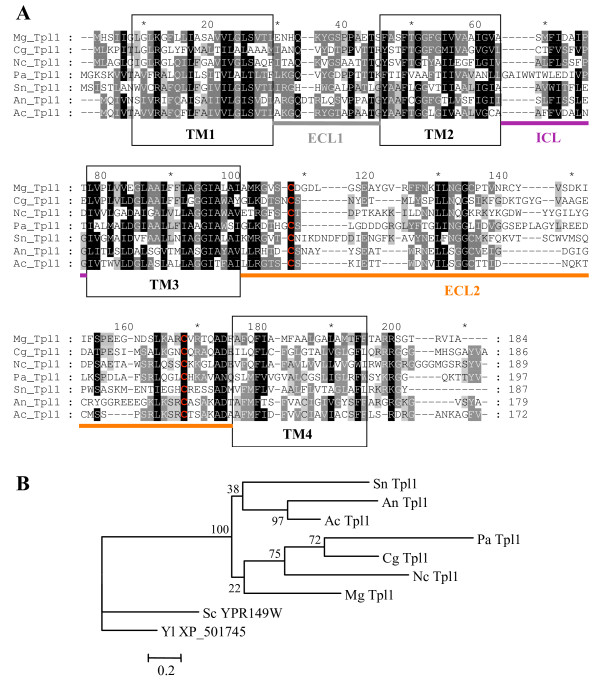
**Alignment and phylogenetic tree of Tpl1 tetraspanins like**. A. Alignment of Tpl1 tetraspanins. Sequences were aligned using ClustalX 1.8. Conserved amino acids are indicated in black (>80%), dark gray (>60%) and light gray (>40%). The transmembrane domains (TM) are circled in black, the small extracellular loop (ECL1), the small intracellular loop (ICL), the large extracellular loop (ECL2) are shown in gray, purple and orange lines, respectively. ECL2 contains two conserved cysteine residues (red). Mg: *Magnaporthe grisea*, Cg: *Chaetomium globosum*, Nc: *Neurospora crassa*, Pa: *Podospora anserina*, Sn:*Stagonospora nodorum*, An: *Aspergillus nidulans*, Ac: *Aspergillus clavatus*. (For a structural model, see Figure 1). B. Phylogenetic tree of fungal Tpl1 proteins. Aligned sequences were analyzed using maximum likelihood from PHYML and XP_501745 from *Yarrowia lipolytica *and Nce2 (YPR149W) from *Saccharomyces cerevisiae *as outgroups. Bootstraps values are expressed as percentage of 100 replicates.

In addition to the tetraspanins described above, the *M. grisea *genomecontains a tetraspanin-like gene, which we have named *TPL1 *(MGG_08113.5). This gene was identified by use of the tetraspanin HMM profile (PF00335) not only in *M. grisea *but also in other ascomycetes such as *C. globosum*, *S. nodorum*, *N. crassa*, *P. anserina *and *A. nidulans *(Figure 4). In marked contrast, the Tpl1 protein is absent from basidiomycete genomes. The MgTPL1 cDNA was reconstructed from ESTs and used to define introns and start/stop codons. *MgTPL1 *has two introns at position 40 and 262 bp from ATG [see Additional file 2]. Tpl1 proteins display some structural hallmarks of tetraspanins such as the presence of four transmembrane domains, a small ECL1 loop (14 aa), a short ICL loop (6–12 aa), a large ECL2 loop (56–68 aa) and a C-terminal cytoplasmic tail (9–17 aa) similar in size to that of Pls1 tetraspanins (Figures 2 and 4, [Additional file 2]). However, Tpl1 proteins markedly differ from Pls1, Tsp2 and Tsp3 tetraspanins in that they lack the typical cysteine based-pattern in the ECL2. Instead, they have two conserved cysteine residues close to the TM3 and TM4, respectively (Figures 2 and 4). For this reason, we have classified these proteins as tetraspanin-like.

### Phylogeny of fungal tetraspanins reveals paralogs only in the Tsp2 family

**Figure 5:**
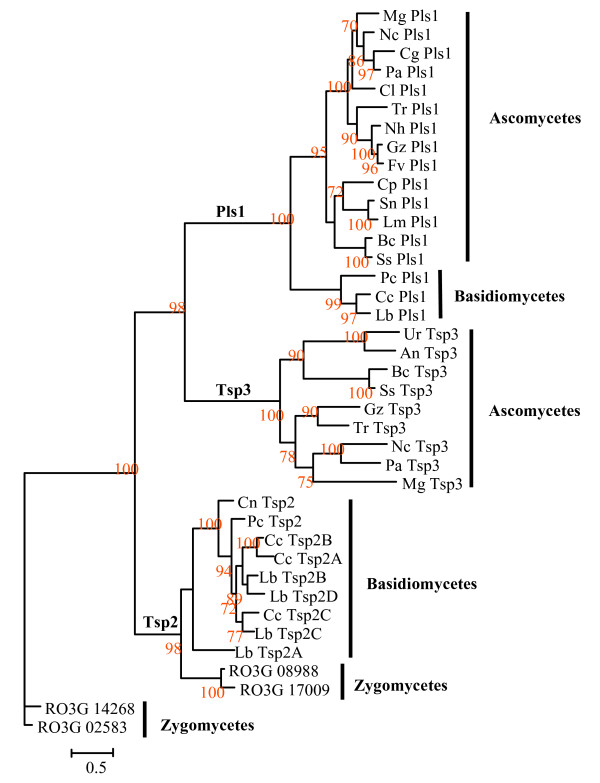
**Phylogenetic tree of fungal Pls1, Tsp2 and Tsp3 tetraspanins**. The Pls1, Tsp2 and Tsp3 were aligned using ClustalX 1.8. Aligned sequences were analyzed by maximum likelihood using PHYML and RO3G_14268 and RO3G_02583 from *R. oryzae *as outgroups [see Additional files 4]. Bootstraps values are expressed as percentage of 100 replicates. Mg: *Magnaporthe grisea*, Cg: *Chaetomium globosum*, Cl: *Colletotrichum lindemuthianum*, Nc: *Neurospora crassa*, Pa: *Podospora anserina*, Nh: *Nectria haematococca*, Gz: *Gibberella zeae*, Fv: *Fusarium verticilloides*, Tr: *Trichoderma reesei*, Sn: *Stagonospora nodorum*, Lm: *Leptosphaeria maculans*, Cp: *Coccidioides posadasii*, Bc: *Botrytis cinerea*, Ss: *Sclerotinia sclerotiorum*, An: *Aspergillus niger*, Lb: *Laccaria bicolor*, Cc: *Coprinus cinereus*, Pc: *Phanerochete chrisosporium*, Cn: *Cryptococcus neoformans*, Ur:*Uncinocarpus reesii*.

Protein sequences from Pls1, Tsp2 and Tsp3 families were aligned using ClustalX 1.8 [see Additional file 4]. A phylogenetic analysis was then conducted using the PHYML software. The Tpl1 tetraspanin-like family was excluded from this alignment as these proteins are too divergent from Pls1, Tsp2 and Tsp3 proteins to be aligned correctly. The resulting phylogenetic tree (Figure 5) shows that Pls1, Tsp2 and Tsp3 form three distinct families. This phylogenetic tree also shows that the *PLS1 *genes from ascomycetes and basidiomycetes are orthologs, as the tree is congruent to the corresponding species phylogeny [20]. Similarly, *TSP3 *genes are orthologs (Figure 5). The *TSP2 *family consists of numerous paralogs, some being recent as their closest relative gene is in the same species (Tsp2B and Tsp2D from *L. bicolor*). The Tsp2 cluster is rooted by tetraspanins in the zygomycete, *R. oryzae *(RO3G_08988/RoTsp2-A and RO3G_17009/RoTsp2-B). The presence of tetraspanins in *R. oryzae *shows that this family of tetraspanins is ancient and predates the split between zygomycetes and higher fungi.

The number of introns in *PLS1 *genes varies from one to three in ascomycetes and from three to four in basidiomycetes [see Additional file 2]. One intron position is conserved in all *PLS1 *genes from ascomycetes (position 403, [see Additional file 2]) except in the related *BcPLS1 *and *SsPLS1 *(Leotiomycetes). This intron conservation supports the orthology found between *PLS1 *genes from ascomycetes. In *PLS1 *from basidiomycetes, three of the four intron positions are conserved [see Additional file 2] confirming a common origin for these three genes. The number of introns in genes from the *TSP2 *family varies from zero to four. However closely related *TSP2 *genes such as *CcTSP2A, CcTSP2B, LbTSP2B *and *LbTSP2D *(60% identity at the protein level) that cluster as a single clade in the phylogenetic tree (Figure 5) share the 2 among three introns, suggesting that they result from recent duplications in both *C. cinereus *and *L. bicolor g*enomes. In Sordariaceae, *TSP3 *genes contain three introns [see Additional file 2] except *N. crassa *which has four, although this may be due to an incorrect exon/intron annotation in the *N. crassa *gene that could not been corrected since it has no ESTs. Other *TSP3 *genes have three conserved intron positions (positions 31–52, 60–81 and 565–601, [see Additional file 2]) except in the related *BcPLS1 *and *SsPLS1 *(Leotiomycetes), which have an additional specific intron. This conservation of intron number and position supports the orthology between *TSP3 *genes.

### Expression of fungal genes encoding tetraspanins

**Figure 6:**
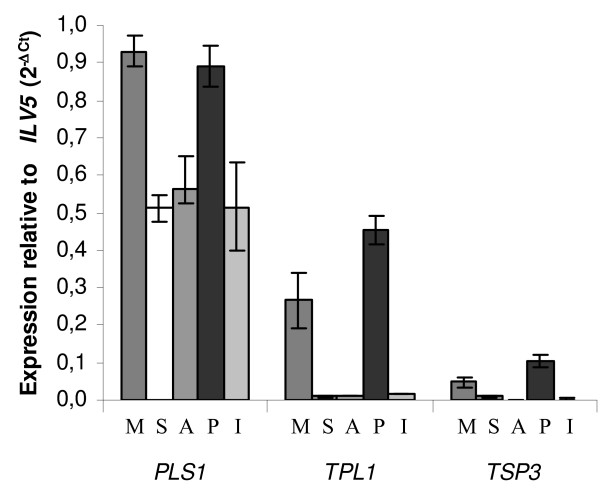
**Expression profiles of *PLS1, TSP3*, *TPL1 *in different tissues from *M. grisea***. *PLS1*, *TSP3*, *TPL1 *expressions were quantified by real-time RT-PCR using RNA extracted from mycelia (M), spores (S), 24-h old appressoria (A) from P1.2 wild type strain, perithecia (P) from crosses between P1.2 (*MAT1.2*) and TH12 (*MAT1.1*) strains and 3-days old barley leaves infected by P1.2 (I). *PLS1*, *TSP3*, *TPL1 *expressions were calculated relative to the transcripts levels of the constitutively expressed gene *ILV5 *(MGG_01808.5) according to the formulae: 2^-ΔCt ^= 2^-(*CtgeneX*-*CtILV*5)^. Each data point is the average of three biological replicates. Standard deviation is indicated by error bars. *PLS1 *is expressed in all tissues whereas *TSP3 *and *TPL1 *are expressed in mycelia and perithecia.

**Figure 7:**
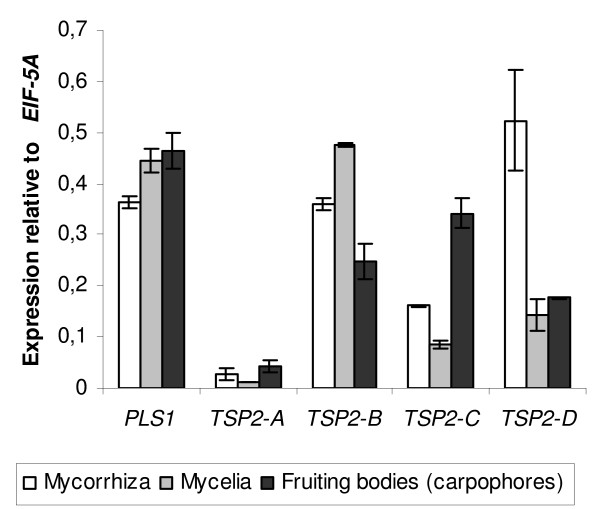
**Expression profiles of *LbPLS1*, *LbTSP2-A*, *LbTSP2-B*, *LbTSP2-C *and *LbTSP2-D *in different tissues from *Laccaria bicolor*. ***LbPLS1 *and *LbTSP2-A*, *LbTSP2-B*, *LbTSP2-C *and *LbTSP2-D *expressions were measured by 60-mer oligoarrays (NimbleGen) using RNA extracted from mycorrhiza, mycelia and fruiting bodies (carpophores). *LbPLS1, LbTSP2-A*, *LbTSP2-B*, *LbTSP2-C *and *LbTSP2-D *expressions were calculated relative to the transcripts levels of the constitutively expressed gene *EIF-5A *(LACBI1_192615). Each data point is the mean between values of two biological replicates and a third value corresponding to the mean of two biological replicates. Standard deviation is indicated by error bars. *PLS1 *is constitutively expressed in all tissues, *LbTSP2-A *transcripts are barely detectable in comparison to *LbTSP2-B*, *LbTSP2-C *and *LbTSP2-D *transcripts that are expressed in all tissues. *LbTSP2-B *is strongly expressed in mycorrhiza and mycelia whereas *LbTSP2-C *and *LbTSP2-D *are up-expressed in fruiting bodies and in mycorrhiza, respectively.

The expression patterns of *PLS1*, *TSP2*, *TSP3 *and *TPL1 *were evaluated *in *different fungal species in *silico *(absence or presence of ESTs from various libraries, [see Additional file 2]), and by quantitative PCR in *M. grisea *for *MgPLS1, MgTSP3 *and *MgTPL1 *(Figure 6) or by microarrays in *L. bicolor *for *LbPLS1*, *LbTSP2-A, LbTSP2-B, LbTSP2-C and LbTSP2-D *(Figure 7).

ESTs corresponding to genes from the *PLS1 *family were identified [see Additional file 2] in *M. grisea *(perithecia-sexual fruiting bodies), *T. reesei*,*B. cinerea *(mycelia), *S. sclerotiorium *(mycelia, sclerotia, and apothecia-sexual fruiting bodies), *Gibberella moniliformis *(mycelia), *P. anserina *(perithecia-sexual fruiting bodies, germinating ascospores, mycelia),*C. cinerea *(mycelia, sexual fruiting bodies), *P. chrysosporium *(tissue undetermined) and *L. bicolor *(free-living mycelia andmycorrhiza). In all these fungi *PLS1 *genes are expressed, ruling out the possibility that they correspond to pseudogenes. Indeed *PLS1 *cDNAs of *L. maculans, S. sclerotiorium *and *C. posadasii *were obtained by the screening cDNA libraries. qPCR expression profiling in *M. grisea *revealed that *MgPLS1 *mRNAis expressed at similar levels in mycelia and perithecia (0.9 × reference constitutive gene *ILV5*, Figure 6) and in spores, appressoria and infected barley leaves (0.5 × *ILV5*, Figure 6). In *L. bicolor*, *PLS1 *is constitutively expressed in all tissues analyzed (mycorrhizal symbiotic tissues, mycelia and fruiting bodies, Figure 7).

The expression patterns of the different *TSP2 *genes in *L. bicolor *were determined using genome wide long oligonucleotide microarrays (Figure 7). *LbTSP2-A *shows a barely detectable expression level in contrast to *LbTSP2-B, LbTSP2-C *and *LbTSP2-D*, which are expressed in all tissues of *L. bicolor*. *LbTSP2-B *is highly expressed in mycorrhiza and mycelia and at a lower level in fruiting bodies. Transcripts corresponding to *LbTSP2-C *are mainly found in fruiting bodies, whereas *LbTSP2-D *is over-expressed in mycorrhiza (Figure 7). ESTs corresponding to *TSP2 *were found in *P. chrysosporium*, *C. neoformans*, *C. cinerea *(mycelia, sexual fruiting bodies) and *L. bicolor *(mycelia) suggesting that these genes are expressed in the corresponding species.

ESTs corresponding to *TSP3 *were identified in *G. zeae *(mycelia, infected wheat heads and perithecia-sexual fruiting bodies), *T. reesei *(mycelia), *Trichoderma harzianum *(mycelia), *B. cinerea *(mycelia), *S. sclerotiorium *(apothecia-sexual fruiting bodies) and *A. niger *(mycelia). Quantitative PCR expression profiling in *M. grisea *showed that *MgTSP3 *is only weakly expressed in perithecia (0.1 × *ILV5*) and mycelia (0.05 × *ILV5*), and negligible in spore and appressoria (Figure 6). Spliced *TSP3 *transcripts were detected by RT-PCR using mycelial RNA (data not shown) suggesting that *TSP3 *is indeed expressed in mycelia, although at a very low level.

ESTs for *TPL1 *were only identified in *M. grisea *and *P. anserina *[see Additional file 2]. Quantitative PCR expression profiling in *M. grisea *revealed that *MgTPL1 *is over-expressed in perithecia and mycelia (0.4 × *ILV5 *and 0.3 × *ILV5*, respectively), but its expression is not detected in spores and appressoria (Figure 6).

### Functional analysis of *TSP3 *and *TPL1 *in *M. grisea*

**Figure 8:**
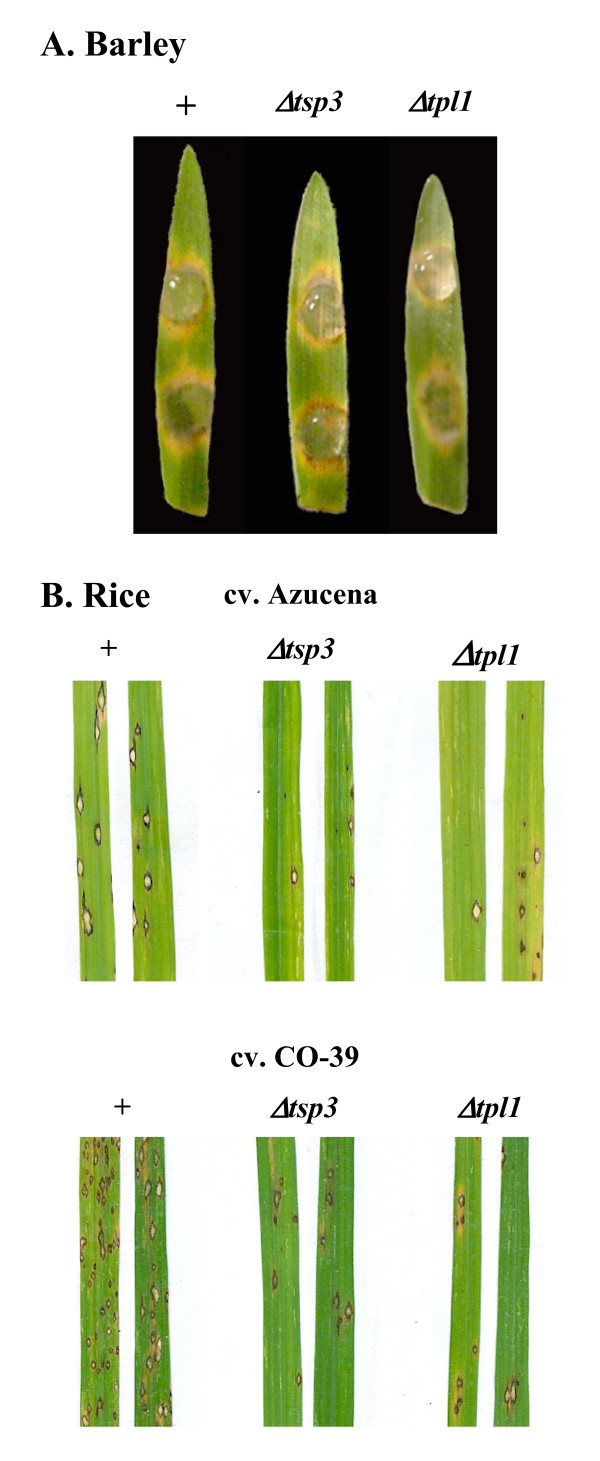
**Pathogenicity of *Δtsp3 *and *Δtpl1 *deletion mutants from *M. grisea***. (A) Pathogenicity on barley. Barley cv. Plaisant leaves were inoculated with droplets of conidial suspension (3 × 10^4 ^conidia/ml) from pathogenic P1.2-*Δku80::bar *(+) and *Δku80::bar/Δtsp3 *and *Δku80::bar/Δtpl1 *mutants. Leaves were kept on water agar for 7 days under alternate day/night at 26°C and scored. Typical symptoms induced by *M. grisea *are visible as pale green ellipsoid lesions surrounded by a yellow halo for wild type as well as tetraspanin mutants showing that *Δtsp3 *and *Δtpl1 *mutants are still pathogenic on barley. (B) Pathogenicity on rice. Seedlings of rice cultivars Azucena and CO-39 were sprayed with conidial suspensions (2.5 × 10^4 ^conidia/ml) from pathogenic P1.2-*Δku80::bar *(+) and *Δku80::bar/Δtsp3 *and *Δku80::bar/Δtpl1 *mutants. Seedlings were incubated for 7 days under alternate day/night at 25°C for development of disease. The deletion mutants *Δku80::bar/Δtsp3 *and *Δku80::bar/Δtpl1 *produced significantly fewer disease lesions than P1.2-*Δku80::bar *(reference strain).

The *M. grisea *deletion mutants of *TSP3 *and *TPL1 *were obtained by targeted gene replacement in a P1.2-*Δku80::bar *mutant background that increases the frequency of homologous recombination [21]. Fifteen and 11 hygromycin-resistant transformants of *TSP3 *and *TPL1*, respectively, were isolated andanalyzed by PCR for the replacement of their wild-type alleles. Deletion mutants were obtained with an efficiency of 100% (15/15) and 82% (9/11) for *TSP3 *and *TPL1*, respectively. Their mycelial growth and sporulation rates were similar to those of P1.2-*Δku80::bar *(reference strain). These mutants were inoculated on detached barley leaves using droplets of conidial suspensions or on barley plants by spraying. Mutants *Δtsp3::hyg *or *Δtpl1::hyg *caused foliar lesions identical in number, size and aspect to those induced by the reference strain P1.2-*Δku80::bar*. The penetration frequencies of thesemutants on barley epidermis were similar to those of wild type, suggesting that *TSP3 *and *TPL1 *are not involved in pathogenicity on barley (Figure 8A). This behavior differs from *Δpls1 *mutant which is non-pathogenic on barley [3]. These mutants were spray-inoculated on two different rice cultivars compatible with *M. grisea *isolate P1.2, Azucena (*O. sativa japonica*) with an intermediate level of partial resistance to *M. grisea *and CO-39 (*O. sativa indica*) that is more susceptible to *M. grisea *than Azucena. Quantitative analysis of leaf blast infection revealed that the pathogenicity of *Δtsp3::hyg *and *Δtpl1::hyg *deletion mutants is significantly reduced on both cultivars. On cv. Azucena, the *Δtsp3::hyg *and *Δtpl1::hyg *mutants produced respectively less than 30% (z = 5, P = 0) and 35% (z = 4.6, P = 2.3 × 10^-6^) of the number of foliar lesions induced by the P1.2-*Δku80::bar *reference strain (Figure 8B). On cv. CO-39, the *Δtsp3::hyg *and *Δtpl1::hyg *mutants produced respectively less than 55% (t = 3.2, P = 1.2 × 10^-3^) and 40% (z = 4.3, P = 8.6 × 10^-6^), respectively, of the number of foliar lesions produced by the P1.2-*Δku80::bar *strain (Figure 8B).

The up-regulation of *TSP3 *and *TPL1 *in perithecia suggested involvement of these genes in mating. To address this question, the differentiation of perithecia and ascospores was examined in crosses between wild-type TH12 isolate and *Δtsp3::hyg *or *Δtpl1::hyg *mutants. A cross between the P1.2-*Δku80::bar *mutant (phenotypically similar to the wild-type P12 isolate) and wild-type TH12 isolate was used as control. Production of perithecia was monitored and three weeks after mating and those ascospores were observed and then allowed to germinate. No significant differences were observed in the fertility in crosses between *Δtsp3::hyg*, *Δtpl1::hyg *and wild type *M. grisea *strains, indicating that these genes are not essential for sexual reproduction.

## Discussion

We have performed an exhaustive search for tetraspanins in fungal genomes. In addition to expanding the previously described Pls1 [11] and Tsp2 tetraspanin families [2], we have identified a new tetraspanin family (Tsp3) and a tetraspanin-like family (Tpl1). These gene families display a different evolutionary history. An ancestral tetraspanin family specific to zygomycetes (RO3G_02583 and RO3G_14268 used as roots of the tree; Figure 5) appears to be lost in higher fungi (basidiomycetes and ascomycetes). The identification of a Tsp2 tetraspanin in the zygomycete *Rhizopus oryzae *(RO3G_08988/RoTsp2-A and RO3G_17009/RoTsp2-B) shows that this family is ancient and predates the divergence between this phylum and higher fungi. Pls1-encoding genes were identified in almost all ascomycete and basidiomycete species. Their absence in zygomycetes suggests that the Pls1 family is more recent than the Tsp2 one (Figure 5). The Tsp3 and Tpl1 families are restricted to ascomycetes suggesting a more recent origin than the Pls1 and Tsp2 families. The absence of tetraspanins in all hemiascomycete species suggests that these genes were lost early after the divergence of these fungi from the other ascomycetes. In the *Aspergilli*, the unique tetraspanin identified belongs to the Tsp3 family. Interestingly no tetraspanin genes were found in the basidiomycete, *U. maydis*.

The Pls1 family is highly conserved in higher fungi and is present as a single copy in each genome, defining a family of orthologous genes. In phytopathogenic fungi, Pls1 is essential for infection and is required for appressorium-mediated penetration into host plant in three species (*M. grisea*, *B. cinerea*,*C. lindemuthianum*). The corresponding null mutants have appressoria that are unable to form functional penetration pegs that direct the penetration of the fungus into host plant [3,13,14]. These results support the hypothesis that Pls1 tetraspanins are involved in a conserved cellular function essential for appressorium function. However, the existence of Pls1 in the human ascomycete pathogen *Coccidioides posadasii*, in saprotrophic fungiand in symbiotic basidiomycetes such as ectomycorrhiza suggests that Pls1 plays other cellular roles than appressorium-mediated penetration of host tissues since these fungi do not differentiate appressoria. At present, these functions of Pls1 remain unknown. In fungi, functional studies are just beginning to be applied to discover such functions. Furthermore, the presence of a single copy of *PLS1 *in these fungal genomes offers an advantage in deciphering its cellular functions, compared to animals that have numerous tetraspanins with possible functional redundancy [6].

The Tsp2 tetraspanins are highly conserved suggesting an important functional role in most basidiomycetes although it is curious that the *Ustilago maydis *genome does not contain tetraspanins. Interestingly, this Tsp2 family is multigenic in *L. bicolor *and *C. cinerea*, which contrasts with other fungal tetraspanins families. Indeed, *L. bicolor *displays four Tsp2 paralogs and *C. cinerea *contains three Tsp2 paralogs. The functional significance of this multigene family is unknown. The finding in *L. bicolor *that *LbTSP2B*, *LbTSP2C *and *LbTSP2D *were expressed in all analyzed tissues suggests that corresponding proteins could be involved in the formation of a tetraspanin web, as described in animals [6]. However, differential expression profiles were observed; *LbTSP2B *is over-expressed in mycelia, *LbTSP2C *is over-expressed in fruiting bodies and *LbTSP2D *is over-expressed in mycorrhiza. This tissue specific expression of Tsp2 tetraspanins may explain the redundancy of this family.

Although *TSP3 *and *TPL1 *are over-expressed in perithecia, no particular phenotype was observed in perithecia when either of these genes was mutated. Perithecial production after mating and ascospore germination were identical to the wild type strain. No significant differences in the fertility were observed in crosses between *Δtsp3::hyg*, *Δtpl1::hyg *and wild type *M. grisea *strains. In animals, analysis of mutants with deleted tetraspanins showed no effect on development, but often had slightly altered phenotypes. For example, deletions of murine tetraspanin genes led to a mild alteration of lymphocyte proliferation and motility [6]. Even, in the Drosophilae the deletion of about 25% of all tetraspanins does not affect viability or fertility and results only in a transient defect in neuromuscular innervation during the larval stage [22]. *M. grisea *may not be an appropriate model to characterize sexual reproduction and anastomosis. Therefore, it would be interesting to investigate the role of the *TSP3 *and *TPL1 *genes in other 'model' fungi such as *N. crassa *or *P. anserina*, which have large sexual fruiting bodies and higher reproduction frequency, and thus may be more suitable to analyze subtle phenotypes.

In *M. grisea*, the *Δtsp3 *and *Δtpl1 *mutants showed a significant reduction in pathogenicity only on rice. The rice cultivars used in this study are generally more resistant to *M. grisea *compared to the barley cultivars. Thus, barley may not be resistant enough to reveal differences in pathogenicity between the *Δtsp3 *and *Δtpl1 *mutants and a wild type strain. Our findings show that *TSP3 *and *TPL1 *are involved in the pathogenicity of *M. grisea *but have only a quantitative effect on pathogenicity, in contrast to the loss of pathogenicity caused by deletion of *PLS1*.

## Conclusion

Higher fungi contain three canonical tetraspanin families: the Pls1 family identified in ascomycetes and basidiomycetes, the Tsp2 family specific to basidiomycetes and the Tsp3 family restricted to ascomycetes. Paralogs were only identified within the Tsp2 family in the basidiomycetes *C. cinereus *and *L. bicolor*. A tetraspanin-like family (Tpl1) was also identified although only in ascomycetes. Our results demonstrate that the deletion of *TSP3 *and *TPL1 *reduced the pathogenicity of *M. grisea *on rice. At present, functional studies of fungal tetraspanins are limited to phytopathogenic fungi producing appressoria. The function(s) of tetraspanin remain(s) unknown in saprophytic fungi, basidiomycetes and human pathogenic fungi. Future functional study of tetraspanins in these fungi may highlight other cellular roles than in appressorium-mediated penetration in host plants. Finally, the role of tetraspanin-like proteins in fungi needs to be investigated in more depth. For example, the possibility of functional interactions with canonical tetraspanins should be assessed as observed in animals [23].

## Methods

### Bioinformatic analyses

Fungal proteins related to tetraspanins were identified using the PF00335 motif [24] and HMMER package [25,26]) to search fungal protein databases available at [27-31]. ESTs were searched by Blastn of the corresponding tetraspanin encoding genes against public databases available at NCBI [29], COGEME [32], Broad Institute [27] or private EST databases (P. Silar, IGM, Orsay, France for *Podospora anserina*; *Laccaria bicolor *database [33]; and Bayer CropScience for *Botrytis cinerea*).

### cDNA libraries

The *PLS1 *cDNA from *S. sclerotiorum *(isolate UQ1280-1) was amplified by PCR using the degenerate primers T1b and T3b [11] from appressorial cDNA prepared as described by [34] and RACE (5' and 3') allowed the sequence of full length transcripts to be acquired. The complete sequence of *PLS1 *from *L. maculans *was derived from a partial EST (DT932772) and by 5' and 3' RACE. The RACE libraries (Invitrogen GeneRacer kit, CA) were constructed using RNA extracted from mycelia grown for four days on vegetable juice medium (10% Campbell's V8 juice, pH 6) in shaking cultures. To identify introns and UTRs in the tetraspanin-encoding genes, BLAST2SEQ [29] and ClustalW were used to align the genomic sequence of each tetraspanin locus with available ESTs. The *PLS1 *cDNAs of *C. posadasii *were isolated by RT-PCR using total RNA from mycelium of strain Silveira as a template. Oligos OAM770 (CGTGACATACCGCTGAATTG) and OAM772 (TATTTGGAATCAACCGCCTC) were used to amplify the cDNA, which was cloned into pGEM^®^-T Easy (Promega) and sequenced. RT-PCR analysis of the CpPLS1 gene using RNA isolated from in vitro grown parasitic phase spherules indicated that it was expressed at all stages of spherulation, as well as during the mycelial phase of the fungus. The various genes and their corresponding proteins are listed in [Additional file 5] and [Additional file 6].

Transmembrane helices were predicted using the TMHMM2.0 server [35,36]. The presence of the CCG-X-C- X-C motif was sought in the large extracellular loop (ECL2) of each protein. Protein sequences were aligned with ClustalX 1.8 [37] and transferred to GenDoc for visualization [38]. Phylogenetic trees were constructed from these alignments using the maximum likelihood method and the PHYML software [39,40] and transferred to Mega3.1 for visualization [41]. Bootstrap values were expressed as percentage of 100 replicates.

### Fungal strains, growth conditions and *Δtsp3*::hyg and *Δtpl1*::hyg deletion mutants

Media composition, maintenance of *M. grisea *cultures, transformation, and sexual crosses were as described by [42,43]. *M. grisea *P1.2 and TH12 strains have been previously described [3,44], as has the P1.2-*Δku80 *strain [21]. Appressoria were differentiated on Teflon membranes (FP301050 Goodfellow, Cambridge, U.K.). The *Δtsp3 *and *Δtpl1 *mutants were constructed by targeted gene replacement of ORFs by a hygromycin resistance cassette as described in [21]. The primers used in all experiments are listed in [Additional file 7].

### Microarray experiments in *Laccaria bicolor*

The *Laccaria *whole-genome expression array manufactured by NimbleGen (Madison, WI) contains in duplicates eight independent, non-identical, 60-mer probes per whole gene model. Included in the microarray are 20, 614 annotated gene models (genome sequence v1.0), 1, 680 additional predicted gene models, 30, 000 random 60-mer control probes and labeling controls. A manuscript fully describing the array is in preparation. Free-living mycelium of *L. bicolor *S238N was grown onto cellophane-covered agar plates containing Pachlewski medium, and was grown for three weeks before harvesting the proliferating hyphal tips at the periphery. Ectomycorrhizas of *L. bicolor*/Douglas fir and *L. bicolor*/Poplar were synthesized in greenhouse experiments. In addition, ectomycorrhizas of *L. bicolor*/Poplar were synthesized using an *in vitro *system. Fruiting bodies of *L. bicolor *S238N were collected below Douglas fir seedlings grown in a greenhouse and inoculated using *L. bicolor *S238N. Tissues were immediately frozen in liquid nitrogen and RNA extraction was carried out using the RNeasy Plant Mini Kit (Qiagen). Total RNA preparations (two biological replicates for each sample) were amplified using the SMART PCR cDNA Synthesis Kit (Clontech) according to the manufacterer's instructions. Single dye labeling of samples, hybridization procedures, data acquisition, background correction and normalization were performed at the NimbleGen facility (NimbleGen Systems, Reykjavik, Iceland) following their standard protocol. Average expression levels were calculated for each gene from the independent probes on the array and were used for further analysis. The complete expression dataset is available as series GSE9784 at the Gene Expression Omnibus at NCBI [45].

### Expression profiling by real-time quantitative PCR

Total RNA was extracted from *M. grisea *mycelia, conidia, mature appressoria (24 h), perithecia and infected barley leaves using the hot acid/phenol protocol [46]. Mycelia were derived from cultures grown in complete liquid medium. Conidia from P1.2 strain were obtained on agar rice medium. To differentiate appressoria, a conidial suspension (3.10^5 ^conidia/mL) supplemented with 10 μM 1,13-hexadecendiol was placed on Teflon membranes (FP301050 Goodfellow, Cambridge, U.K.) and incubated for 24 h at 26°C. Perithecia were obtained by crossing *M. grisea *strains P1.2 and TH12. Reverse transcriptase-polymerase chain reactions (RT-PCR) were performed with 5 μg total RNA using the ThermoScript™ RT-PCR system kit (Invitrogen, Carlsbad, CA) according to the manufacturer' s instructions. Real-time PCR experiments were performed in 96-well plates using ABI-7900 (Applied Biosystems, USA). Primer pairs were designed using Primer Express software (Applied Biosystems, Foster City, CA) with standard parameters (optimal melting point, 58°C; optimal primer length, 20 bp; amplicon length, 101 bp). cDNA dilutions of 10^-3 ^and 10^-2 ^were used respectively for cDNA obtained from fungal tissues (mycelia, conidia, appressoria, and perithecia) and infected barley leaves. Standard conditions were used with SYBR green PCR Master mix (Applied Biosystem, 95°C for 10 min and 40 cycles of 95°C for 30 s, 60°C for 1 min and 72°C for 30 s). Gene expression was calculated relative to the transcripts levels of the constitutively expressed gene *ILV5 *(MGG_01808.5) using the formulae 2^-ΔCt ^= 2^-(*Ctgene*-*CtILV*5)^. The accession numbers of fungal tetraspanins are indicated in [Additional file 5] and the real-time PCR primers are listed in [Additional file 7].

### Phenotypic analysis

Pathogenicity assays were performed using susceptible barley cultivar Plaisant (*Hordeum vulgare *L.) and the susceptible rice cultivars Maratelli, LS1, Azucena and CO-39 (*Oriza sativa*). Conidia harvested from 10–14 day old cultures of *M. grisea *growing on rice agar were inoculated on barley or rice plants. Barley seedlings were cultivated at 15°C with 60% humidity for 2–3 weeks. Segments (3 cm long) from the barley leaves were placed on water agar (1%) containing kinetin (2 mg.L-1), inoculated with 50 μL droplets of spore suspension (3 × 10^4 ^spores.mL^-1^) and incubated at 26°C under a photoperiod of 12 hours light. Leaf symptoms were recorded 7 days after inoculation. Rice seedlings were cultivated for 20–30 days at 70% relative humidity and 25°C day/20°C night in a growth chamber. Barley or rice (five and 160 plants per pot or tray, respectively) were sprayed with spore suspensions (20 mL of 3 × 10^4^and 2.5 × 10^4 ^spores.mL^-1^, respectively) containing 0.5% gelatin (w/v). Inoculated rice plants were grown at 100% humidity for 24 h at 20–22°C in the dark and subsequently transferred to a greenhouse at 23°C. Leaf symptoms were recorded 7 days after inoculation. Quantitative analysis of leaf blast infection was carried on 30 leaves in two biological replicates. Mean number of lesions per leaves were recorded. Statistical analyses were carried out with Statbox Pro 6.5 (Grimmersoft, Paris, France; Student and Mann-Whitney tests, threshold alpha/2 = 0,025). The *M. grisea *penetration assays into barley epidermal cells were performed as described in [3].

## Authors' contributions

KL contributed to the design and analysis of the study, identified tetraspanins (including Tsp3 and Tpl1) in fungal genomes, carried out the functional analysis of Tsp3 and Tpl1 in *M. grisea *and drafted the manuscript. DT analyzed the *M. grisea Δtsp3 *and *Δtpl1 *mutants for their fertility in crosses and their pathogenicity on rice. AK and FM identified tetraspanins and performed microarray experiments in *Laccaria bicolor*. CS contributed to the bioinformatic analyses of fungal genomes. MM helped to the analysis of *M. grisea Δtsp3 *mutant. CB contributed to the expression analysis of tetraspanin-encoding genes in *M. grisea*. ACS and BJH cloned the *PLS1 *cDNA and annotated *PLS1 *from *Leptosphaeria maculans *and *Sclerotinia sclerotiorum*. EMK and MJO cloned the *PLS1 *cDNA and annotated *PLS1 *from *Coccidioides posadasii*. MHL contributed to the design and analysis of this study.

## Supplementary Material

Additional File 1 **Alignment of Pls1 tetraspanins**. Sequences were aligned using ClustalX 1.8. Conserved amino acids are indicated in black (>80%), dark gray (>60%) and light gray (>40%). The transmembrane domains (TM) are circled in black, the small extracellular loop (ECL1), the small intracellular loop (ICL), the large extracellular loop (ECL2) are shown in gray, purple and orange lines, respectively. ECL2 contains a CCG motif and further two conserved cysteine residues (red). These cysteine residues are involved in the formation of two disulphide bridges crucial for ECL2 folding. The conserved polar/charged amino acids in TMs are indicated in blue. One putative palmitoylation site is located proximal to TM4 (green arrow). Mg: *Magnaporthe grisea*, Cg: *Chaetomium globosum*, Cl: *Colletotrichum lindemuthianum*, Nc: *Neurospora crassa*, Pa: *Podospora anserina*, Nh: *Nectria haematococca*, Gz: *Gibberella zeae*, Fv: *Fusarium verticilloides*, Tr: *Trichoderma reesei*, Sn: *Stagonospora nodorum*, Lm: *Leptosphaeria maculans*, Cp: *Coccidioides posadasii*, Bc: *Botrytis cinerea*, Ss: *Sclerotinia sclerotiorum*, Lb: *Laccaria bicolor*, Cc: *Coprinus cinereus*, Pc: *Phanerochete chrisosporium*. For a tetraspanin structural model, see Figure 1. Click here for file

Additional File 2 **Structure of fungal tetraspanin genes and their predicted proteins**. The intron positions are in bp (base pair) from start codon for Pls1, Tsp3 and Tpl1, and from the first transmembrane domain for Tsp2. The number of EST identified for fungal tetraspanins are indicated independently of the source. The amino acids number of the N-terminal tail, small extracellular loop (ECL1), intracellular loop (ICL), large extracellular loop (ECL2), C-terminal tail and ECL2 cysteine motif are indicated in amino-acids are listed in columns. GA (germinating ascospores), FB (fruiting bodies), I (infected tissue), M (mycelia), MR (Mycorrhizal tissue, poplar), Sc (Sclerotia), nd (not defined), as (antisense), TC (Tentative Consensus sequences originating from ESTs), * cDNA available. # manually annotated gene without available ESTs. Click here for file

Additional File 3 **Alignment of Tsp2 tetraspanins**. Sequences were aligned using ClustalX 1.8. The N terminus sequences located before the first TM are not shown, as they are not enough conserved to be aligned. Conserved amino acids are indicated in black (>80%), dark gray (>60%) and light gray (>40%). The transmembrane domains (TM) are circled in black, the small extracellular loop (ECL1), the small intracellular loop (ICL), the large extracellular loop (ECL2) are shown in gray, purple and orange lines, respectively. ECL2 contains a CCG motif and further two conserved cysteine residues (red). These cysteine residues allow formation of two disulphide bridges crucial for the folding of ECL2. One putative conserved palmitoylation (cysteine residue) site is located at the end of TM4, proximal to the inner side of the membrane according to the predicted fungal tetraspanin topology (green arrow). Additionally, LbTsp2A and PcTsp2 display another putative palmitoylation site (cysteine residue) at the start of TM1 (green arrow). Lb: *Laccaria bicolor*, Cc: *Coprinus cinereus*, Pc: *Phanerochete chrisosporium*, Cn: *Cryptococcus neoformans*. RO3G_17009 and RO3G_08988 are two tetraspanins (Tsp2) from *Rhizopus oryzae*. For a tetraspanin structural model, see Figure 1. #: C-terminal tail no defined. Click here for file

Additional File 4 **Alignment of protein sequences from Pls1, Tsp2 and Tsp3 families used for the construction of tree from **Figure 7. This alignment was carried out using ClustalX 1.8. Conserved amino acids are indicated in black (>60%), dark gray (>40%) and light gray (>20%). Click here for file

Additional File 5 **Accession number of fungal tetraspanins**. * The manual correction gene annotations were performed using ESTs when available and the protein alignments. For the sequences, [see Additional file 6]. Click here for file

Additional File 6 **Tetraspanin nucleotide and protein sequences used for this study**. The introns are indicated in bold and yellow. Click here for file

Additional File 7 **Primers used for this study**. Primers used for genes tested by quantitative RT-PCR and for targeted gene replacement of *TSP3 *and *TPL1 *ORFs in *M. grisea*. Click here for file
