# Supplementary figures and images for: Fungi have three tetraspanin families with distinct functions
Source: BMC Genomics. 2008 Feb 3;9:63. doi: 10.1186/1471-2164-9-63 (PMC2278132; doi:10.1186/1471-2164-9-63)

## Additional file 1

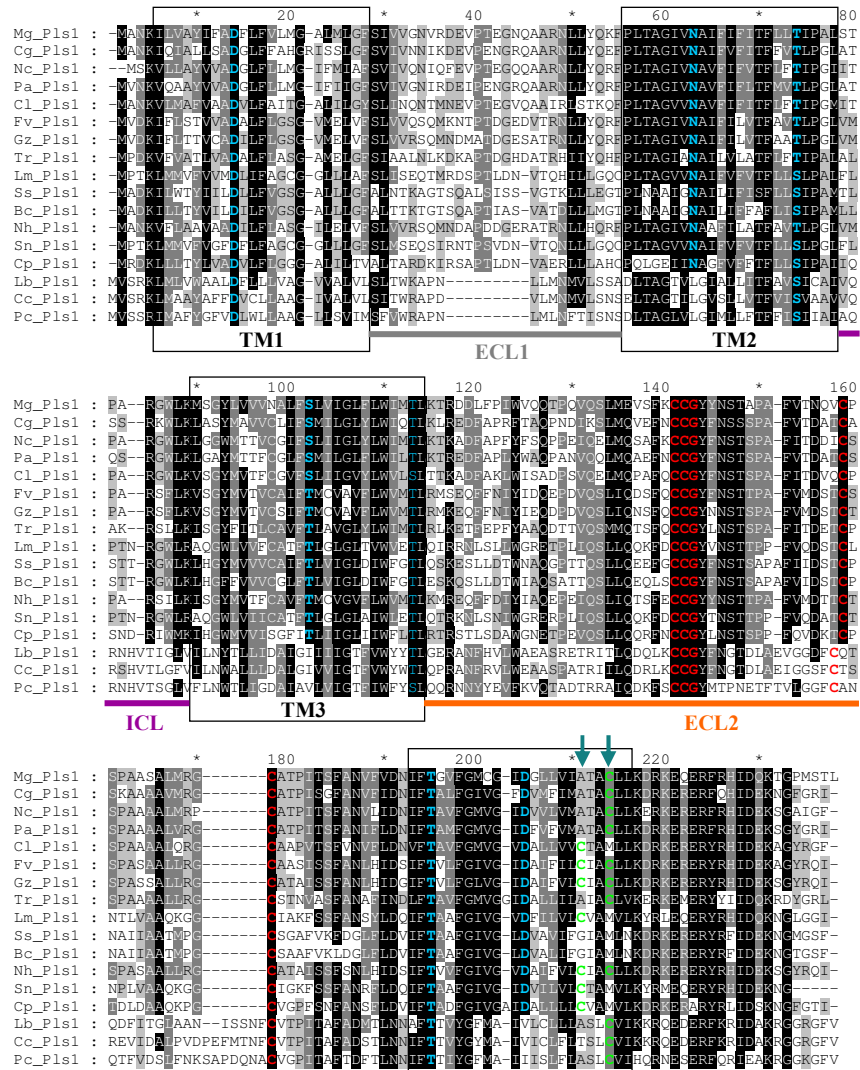

Supplement: Additional File 1 — Alignment of Pls1 tetraspanins. Sequences were aligned using ClustalX 1.8. Conserved amino acids are indicated in black (>80%), dark gray (>60%) and light gray (>40%). The transmembrane domains (TM) are circled in black, the small extracellular loop (ECL1), the small intracellular loop (ICL), the large extracellular loop (ECL2) are shown in gray, purple and orange lines, respectively. ECL2 contains a CCG motif and further two conserved cysteine residues (red). These cysteine residues are involved in the formation of two disulphide bridges crucial for ECL2 folding. The conserved polar/charged amino acids in TMs are indicated in blue. One putative palmitoylation site is located proximal to TM4 (green arrow). Mg: Magnaporthe grisea, Cg: Chaetomium globosum, Cl: Colletotrichum lindemuthianum, Nc: Neurospora crassa, Pa: Podospora anserina, Nh: Nectria haematococca, Gz: Gibberella zeae, Fv: Fusarium verticilloides, Tr: Trichoderma reesei, Sn: Stagonospora nodorum, Lm: Leptosphaeria maculans, Cp: Coccidioides posadasii, Bc: Botrytis cinerea, Ss: Sclerotinia sclerotiorum, Lb: Laccaria bicolor, Cc: Coprinus cinereus, Pc: Phanerochete chrisosporium. For a tetraspanin structural model, see Figure 1. [file 1471-2164-9-63-S1.PDF]

### Additional file 3

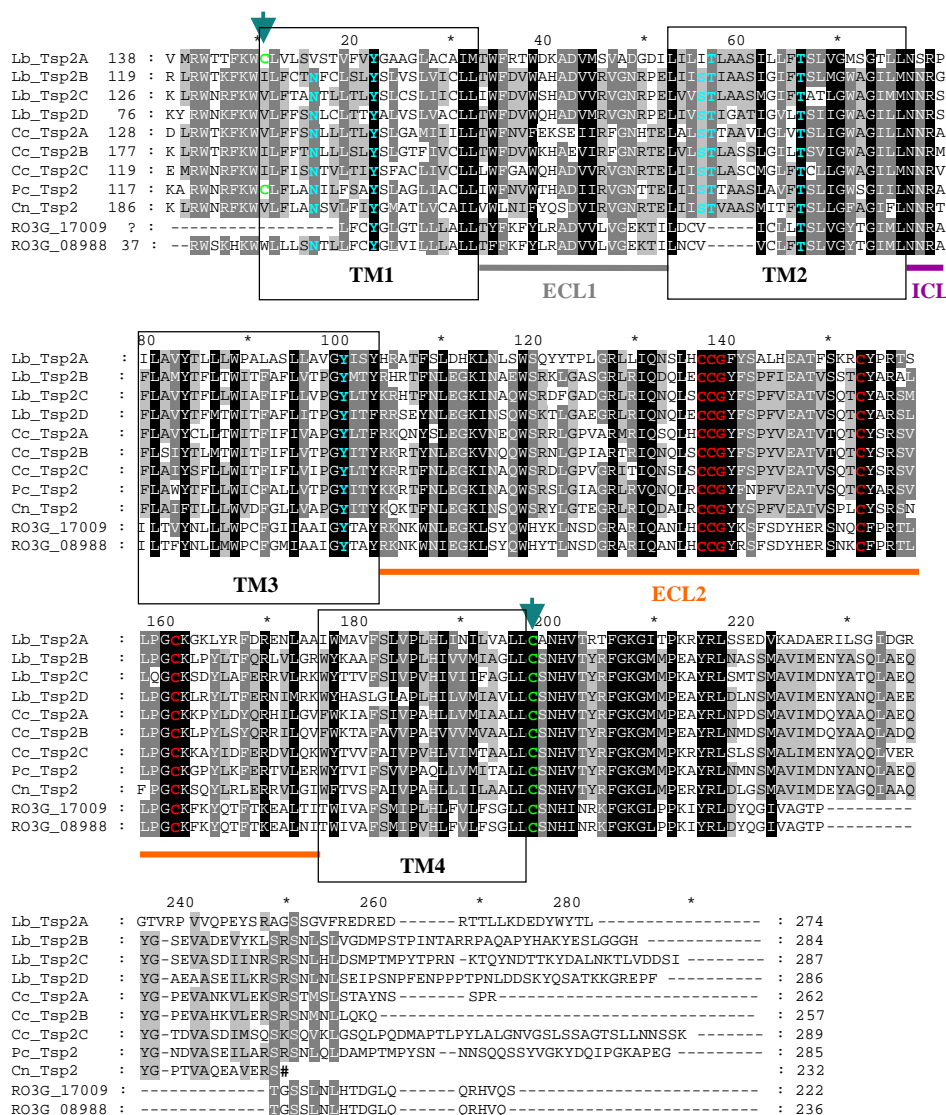

Supplement: Additional File 3 — Alignment of Tsp2 tetraspanins. Sequences were aligned using ClustalX 1.8. The N terminus sequences located before the first TM are not shown, as they are not enough conserved to be aligned. Conserved amino acids are indicated in black (>80%), dark gray (>60%) and light gray (>40%). The transmembrane domains (TM) are circled in black, the small extracellular loop (ECL1), the small intracellular loop (ICL), the large extracellular loop (ECL2) are shown in gray, purple and orange lines, respectively. ECL2 contains a CCG motif and further two conserved cysteine residues (red). These cysteine residues allow formation of two disulphide bridges crucial for the folding of ECL2. One putative conserved palmitoylation (cysteine residue) site is located at the end of TM4, proximal to the inner side of the membrane according to the predicted fungal tetraspanin topology (green arrow). Additionally, LbTsp2A and PcTsp2 display another putative palmitoylation site (cysteine residue) at the start of TM1 (green arrow). Lb: Laccaria bicolor, Cc: Coprinus cinereus, Pc: Phanerochete chrisosporium, Cn: Cryptococcus neoformans. RO3G_17009 and RO3G_08988 are two tetraspanins (Tsp2) from Rhizopus oryzae. For a tetraspanin structural model, see Figure 1. #: C-terminal tail no defined. [file 1471-2164-9-63-S3.PDF]
