# Supplementary material for: Fungi have three tetraspanin families with distinct functions
Source: BMC Genomics. 2008 Feb 3;9:63. doi: 10.1186/1471-2164-9-63 (PMC2278132; doi:10.1186/1471-2164-9-63)
Supplement: Additional File 2 — Structure of fungal tetraspanin genes and their predicted proteins. The intron positions are in bp (base pair) from start codon for Pls1, Tsp3 and Tpl1, and from the first transmembrane domain for Tsp2. The number of EST identified for fungal tetraspanins are indicated independently of the source. The amino acids number of the N-terminal tail, small extracellular loop (ECL1), intracellular loop (ICL), large extracellular loop (ECL2), C-terminal tail and ECL2 cysteine motif are indicated in amino-acids are listed in columns. GA (germinating ascospores), FB (fruiting bodies), I (infected tissue), M (mycelia), MR (Mycorrhizal tissue, poplar), Sc (Sclerotia), nd (not defined), as (antisense), TC (Tentative Consensus sequences originating from ESTs), * cDNA available. # manually annotated gene without available ESTs. [file 1471-2164-9-63-S2.PDF]

Additional file2

		Species	N-term	EC1	ICL	EC2	EC2 motif	C-term	intron position	Number of EST	EST Tissue
Pls1	MgPls1	*M. grisea*	4	26	8	70	CCGY-x(13)-C-x(10)-GC	22	403 569	1	FB
	CgPls1	*C. globosum*	4	26	8	70	CCGY-x(13)-C-x(10)-GC	21	176 409		
	TrPls1	*T. reesei*	4	26	8	72	CCGY-x(13)-C-x(10)-GC	15	403 569	6	
	NcPls1	*N. crassa*	3	26	8	70	CCGY-x(13)-C-x(10)-GC	21	167 400		
	PaPls1	*P. anserina*	4	26	8	70	CCGY-x(13)-C-x(10)-GC	17	403	13	FB, GA, M
	ClPls1	*C. lindemuthianum*	4	26	8	70	CCGY-x(13)-C-x(10)-GC	21	403	*	
	NhPls1	*N. haematococca*	4	26	8	70	CCGY-x(13)-C-x(10)-GC	21	403 569		
	GzPls1	*G. zea*	4	26	8	70	CCGY-x(13)-C-x(10)-GC	21	403 569	12	M
	FvPls1	*F. verticilloides*	4	26	8	70	CCGY-x(13)-C-x(10)-GC	21	403 569		
	SnPls1	*S. nodorum*	4	25	9	70	CCGY-x(13)-C-x(10)-GC	17	167 403		
	CpPls1	*C. posadasii*	4	25	9	70	CCGY-x(13)-C-x(10)-GC	21	167 403 569	*	
	LmPls1	*L. maculans*	4	25	9	70	CCGY-x(13)-C-x(10)-GC	21	167 403	*	
	BcPls1	*B. cinerea*	4	25	9	71	CCGY-x(14)-C-x(10)-GC	21	167	14	M
	SsPls1	*S. sclerotiorum*	4	25	9	71	CCGY-x(14)-C-x(10)-GC	21	167 572	8 *	M, Sc, FB
	CcPls1	*Coprinopsis cinerea*	5	17	10	78	CCGY-x(13)-C-x(19)-C	22	145 559 622	6	M(s), FB (as)
	PcPls1	*P. chrysosporium*	5	17	10	78	CCGY-x(13)-C-x(19)-C	22	146 385 555 622	5	nd
	LbPls1	*Laccaria bicolor*	5	17	10	76	CCGY-x(13)-C-x(17)-C	22	146 385 549 553 616	3 TC (6 ESTs)	M, MR
Tsp2	CnTsp2	*C. neoformans*	200	19	4	72	CCGY-x(12)-CY-x(5)-GCK	88	52 158 397 687	18	nd
	LbTsp2-A	*A-Laccaria bicolor*	292	19	4	72	CCGY-x(12)-CY-x(5)-GCK	85	no intron		
	LbTsp2-B	*B-Laccaria bicolor*	126	19	4	72	CCGY-x(12)-CY-x(5)-GCK	88	(-338) 52 687	2 TC (10 ESTs)	M
	LbTsp2-C	*C-Laccaria bicolor*	133	19	4	72	CCGY-x(12)-CY-x(5)-GCK	93	52 687	1 TC (2 ESTs)	M
	LbTsp2-D	*D-Laccaria bicolor*	78	19	4	72	CCGY-x(12)-CY-x(5)-GCK	91	52 687	1 TC (2 ESTs)	M
	CcTsp2-A	*A_ Coprinopsis cinerea*	102	19	4	72	CCGY-x(12)-CY-x(5)-GCK	67	(-347) 52 687	2	M
	CcTsp2-B	*B_ Coprinopsis cinerea*	184	19	4	72	CCGY-x(12)-CY-x(5)-GCK	62	(-348) 52 687	3	FB
	CcTsp2-C	*C_ Coprinopsis cinerea*	126	19	4	72	CCGY-x(12)-CY-x(5)-GCK	94	687		
	PcTsp2	*P. chrysosporium*	116	19	4	72	CCGY-x(12)-CY-x(5)-GCK	90	(-422) 52 687		
	RoTsp2-A	*Rhizopus oryzae*	88	19	4	72	CCGY-x(12)-CY-x(5)-GCK	48	52 107 158 382	1	nd
	RoTsp2-B	*Rhizopus oryzae*	?	19	4	72	CCGY-x(12)-CY-x(5)-GCK	48	(?)		
Tsp3	MgTsp3	*M. grisea*	3	8	27	81	CCG-x(21)-C-x(9)-C	106	37 66 595	*	M
	NcTsp3	*N. crassa*	1	8	21	81	CCG-x(20)-C-x(10)-C	103	31 188 305 594 #		
	PaTsp3	*P. anserina*	1	8	19	80	CCG-x(22)-C-x(9)-C	98	31 60 568		
	TrTsp3	*T. reesei (TM1et 2)*	2	8	21	79	CCG-x(20)-C-x(9)-C	80	34 63 574	9	M
	GzTsp3	*G. zea*	1	8	21	77	CCG-x(18)-C-x(9)-C	79	31 60 565	6	M, FB, I
	BcTsp3	*B. cinerea*	5	8	21	78	CCG-x(19)-C-x(9)-C	79	43 72 495 580	4	M
	SsTsp3	*S. sclerotiorum*	5	8	21	79	CCG-x(20)-C-x(9)-C	77	43 72 498 583	10	FB
	UrTsp3	*Uncinocarpus reesii*	8	8	23	79	CCG-x(20)-C-x(9)-C	60	52 81 598		
	AnTsp3	*Aspergillus niger*	8	8	23	79	CCG-x(20)-C-x(9)-C	59	52 81 601	2	M
Tpl1	MgTpl1	*M. grisea*	6	14	9	61	SC-x(21)-C-x(5)-C-x(20)-C	11	40 262	4	M
	CgTpl1	*C. globosum*	6	14	9	59	CS-x(45)-C	14	40 262		
	SnTpl1	*S. nodorum*	8	14	9	67	SC-x(26)-C-x(8)-C-x(19)-C	6	268		
	NcTpl1	*N. crassa*	6	14	9	60	SC-x(47)-C	13	40 262		
	PaTpl1	*P. anserina*	6	14	14	64	CS-x(51)-C	11	46 283	2	M
	AnTpl1	*Aspergillus nidulans*	5	16	9	55	CS-x(14)-C-x(8)-C-x(10)-C	10	36 265		
	AcTpl1	*Aspergillus clavatus*	5	14	9	48	CS-x(17)-C-x(8)-C-x(14)-C	12	37 259		
	YlTpl1	*Yarrowia lipolytica*	6	14	11	45	SC-x(19)-C	13	0		
Tsp4	RoTsp4-A	*Rhizopus oryzae*	9	18	9	70	CCGY-x(13)-C-x(12)-C	87	21 155 388	6	nd
	RoTsp4-B	*Rhizopus oryzae*	8	18	9	70	CCGY-x(13)-C-x(12)-C	99	21 149 382		
	PbTsp4	*Phycomyces blakesleeanus*	3	18	9	70	CCGY-x(13)-C-x(12)-C	75	134 367		
