# Supplementary material for: Fungi have three tetraspanin families with distinct functions
Source: BMC Genomics. 2008 Feb 3;9:63. doi: 10.1186/1471-2164-9-63 (PMC2278132; doi:10.1186/1471-2164-9-63)

Additional file 4

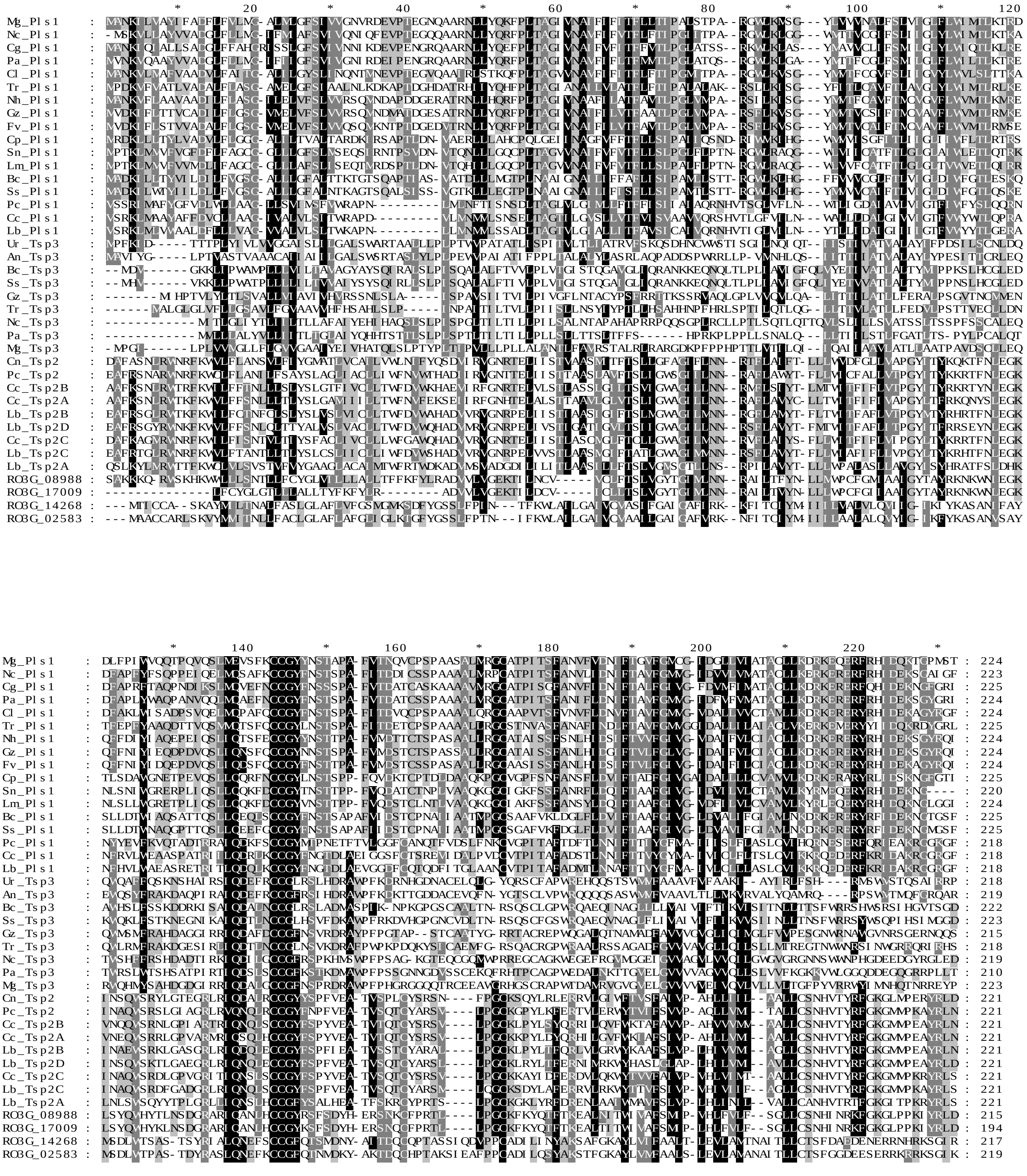

Supplement: Additional File 4 — Alignment of protein sequences from Pls1, Tsp2 and Tsp3 families used for the construction of tree from Figure 7. This alignment was carried out using ClustalX 1.8. Conserved amino acids are indicated in black (>60%), dark gray (>40%) and light gray (>20%). [file 1471-2164-9-63-S4.PDF]
