# Supplementary material for: Fungi have three tetraspanin families with distinct functions
Source: BMC Genomics. 2008 Feb 3;9:63. doi: 10.1186/1471-2164-9-63 (PMC2278132; doi:10.1186/1471-2164-9-63)
Supplement: Additional File 5 — Accession number of fungal tetraspanins. * The manual correction gene annotations were performed using ESTs when available and the protein alignments. For the sequences, [see Additional file 6]. [file 1471-2164-9-63-S5.PDF]

Additional file 5

		Species	Accession number	Source
Pls1	MgPls1	*Magnaporthe grisea*	GenBank: [AX058235]	NCBI [29]
	CgPls1	*Chaetomium globosum*	CHGG_06472 *	Broad institute [27]
	TrPls1	*Trichoderma reesei*	FGENESH1_PM.C_SCAFFOLD_6000072 [trire1:29304]	JGI [28]
	NcPls1	*Neurospora crassa*	GenBank: [AJ504996]	NCBI [29]
	PaPls1	*Podospora anserina*	PA_1_19270	*Podospora* Genome Project [30]
	ClPls1	*Colletotrichum lindemuthianum*	GenBank: [AJ504995]	NCBI [29]
	NhPls1	*Nectria haematococca*	e_gw1.2.1063.1 [Necha2:35986]	JGI [28]
	GzPls1	*Gibberella zea*	FG08695.1	Broad institute [27]
	FvPls1	*Fusarium verticilloides*	FVEG_01966.3	Broad institute [27]
	SnPls1	*Stagonospora nodorum*	SNOG_07760	Broad institute [27]
	CpPls1	*Coccidioides posadasii*	GenBank: [EU374215]	NCBI [29]
	CiPls1	*Coccidioides immitis*	CIMG_05817	Broad institute [27]
	LmPls1	*Leptospheria maculans*	GenBank: [EU090236]	NCBI [29]
	BcPls1	*Botrytis cinerea*	GenBank: [AJ504994]	NCBI [29]
	SsPls1	*Sclerotinia sclerotiorum*	GenBank: [EU090237]	NCBI [29]
	CcPls1	*Coprinopsis cinerea*	CC1G_05148 / GenBank: [EAU88382]	Broad institute [27] /NCBI [29]
	PcPls1	*Phanerochaete chrysosporium*	fgenesh1_pg.C_scaffold_1000644 [Phchr1:644] *	JGI [28]
	LbPls1	*Laccaria bicolor*	fgenesh1_kg.C_scaffold_6000016 [Lacbi1:123030]	JGI [28]
Tsp2	CnTsp2	*Cryptococcus neoformans*	GenBank: [AAW45945]	NCBI [29]
	LbTsp2-A	*A-Laccaria bicolor*	fgenesh3_pg.C_scaffold_3000324 [Lacbi1:322707]	JGI [28]
	LbTsp2-B	*B-Laccaria bicolor*	eu2.Lbscf0012g03050 [Lacbi1:297750]	JGI [28]
	LbTsp2-C	*C-Laccaria bicolor*	eu2.Lbscf0033g00750 [Lacbi1:307020]	JGI [28]
	LbTsp2-D	*D-Laccaria bicolor*	estExt_fgenesh2_pg.C_120287 [Lacbi1:294427] *	JGI [28]
	CcTsp2-A	*A_ Coprinopsis cinerea*	CC1G_11420 / GenBank: [EAU92135]	Broad institute [27] /NCBI [29]
	CcTsp2-B	*B_ Coprinopsis cinerea*	CC1G_09985 / GenBank: [EAU89016]	Broad institute [27] /NCBI [29]
	CcTsp2-C	*C_ Coprinopsis cinerea*	CC1G_04990 / GenBank: [EAU85773]	Broad institute [27] /NCBI [29]
	PcTsp2	*Phanerochaete chrysosporium*	fgenesh1_pg.C_scaffold_13000131 [Phchr1:7343] *	JGI [28]
	RoTsp2-A	*Rhizopus oryzae*	RO3G_08988 */ GenBank: [EU142017]	Broad institute [27] /NCBI [29]
	RoTsp2-B	*Rhizopus oryzae*	RO3G_17009 *	Broad institute [27]
Tsp3	MgTsp3	*Magnaporthe grisea*	MGG_13913.5 */ GenBank: [EU142014]	Broad institute [27] /NCBI [29]
	NcTsp3	*Neurospora crassa*	NCU09755.3 *	Broad institute [27]
	PaTsp3	*Podospora anserina*	GenBank: [EU368756]	NCBI [29]
	TrTsp3	*Trichoderma reesei*	estExt_fgenesh1_pg.C_240020 [trire1:45687]	JGI [28]
	GzTsp3	*Gibberella zea*	FG00962.1	Broad institute [27]
	BcTsp3	*Botrytis cinerea*	BC1G_12905.1	Broad institute [27]
	SsTsp3	*Sclerotinia sclerotiorum*	SS1G_04021 */ GenBank: [EU142016]	Broad institute [27] /NCBI [29]
	UrTsp3	*Uncinocarpus reesii*	UREG_01276	Broad institute [27]
	AnTsp3	*Aspergillus niger*	GenBank: [EU142015]	NCBI [29]
Tpl1	MgTpl1	*Magnaporthe grisea*	MGG_08113.5	Broad institute [27]
	CgTpl1	*Chaetomium globosum*	CHGG_03766.1	Broad institute [27]
	SnTpl1	*Stagonospora nodorum*	SNOG_00997	Broad institute [27]
	NcTpl1	*Neurospora crassa*	NCU05230.2	Broad institute [27]
	PaTpl1	*Podospora anserina*	PA_1_19270	*Podospora* Genome Project [30]
	AnTpl1	*Aspergillus nidulans*	AN8422.3	Broad institute [27]
	AcTpl1	*Aspergillus clavatus*	GenBank: [XP_001276466]	NCBI [29]
	YlTpl1	*Yarrowia lipolytica*	GenBank: [XP_501745]	NCBI [29]
	RoTsp4-A	*Rhizopus oryzae*	RO3G_02583	Broad institute [27]
	RoTsp4-B	*Rhizopus oryzae*	RO3G_14268 * / GenBank: [EU142018]	Broad institute [27] /NCBI [29]
	PbTsp4	*Phycomyces blakesleeanus*	fgeneshPB_pg.3__829 [Phybl1:61204]	JGI [28]
