# Supplementary material for: Fungi have three tetraspanin families with distinct functions
Source: BMC Genomics. 2008 Feb 3;9:63. doi: 10.1186/1471-2164-9-63 (PMC2278132; doi:10.1186/1471-2164-9-63)
Supplement: Additional File 6 — Tetraspanin nucleotide and protein sequences used for this study. The introns are indicated in bold and yellow. [file 1471-2164-9-63-S6.PDF]

Additional file 6

intron

>Lb_Pls1_ _correction of LACBI1_123030_manually annotated gene using EST
atggtctccaggaaactgatgcttgtctgggcagccctagactttcttcttctcgtcgcaggagtggtagctctc
gttctatctctcacatggaaggcccccaatcttctcatgaacatggtcctttcctccgccgatttgactg**gtgtg**
ctgcggtttattgcctttctggtttatttctgattacccgccccttagctggaaccgtgttgggaatagcgttgc
taatcacgtttgcggtttccatatgtgccattgtgcagcggaaccatgtcaccatcggtcttgtcattctcaact
atacccttcttattgatgccatcggcattattattattggcacttttgtttggtattatacccttggagaacgtg
ccaacttccatgtactctgggcggaagcaagccgtgaaactcgtattactctccaggaccag**gtgtgttcctaga**
tattggcaaacccgttcacgacctcatcgatttgttagctcaaatgttgtggatatttcaatggaacagatttgg
ctgaagtcgggggtgattttgtcagactcaagactttatcaccggtcttgcggccaacatctcatccaatttt
gtgtcacgcctatcaccgcctttgctgacatgacgttgaacaacgctttcac**gtatgttgttccatcttgttcct**
aaaatctatactcatctccctctttcagcact**gtatgtcctcttgaacttgctgtggtaaaattattggctgaat**
cgcaatataggtatatggctttatggccatcgtattgtgtttgcttttggcgtcgctgtgtgttatcaagaag**gt**
gaacattttgcatactttaaatcactttctcaccattaattttcagaggcaagaagatgaacgtttcaagagaa
ttgatgccaagcggggtggaagaggtttcgtttaa

>Lb_Pls1_correction of LACBI1_123030_CDS using EST
ATGGTCTCCAGGAAACTGATGCTTGTCTGGGCAGCCCTAGACTTTCTTCTTCTCGTCGCAGGAGTGGTAGCTCTC
GTTCTATCTCTCACATGGAAGGCCCCCAATCTTCTCATGAACATGGTCCTTTCCTCCGCCGATTTGACTGCTGGA
ACCGTGTTGGGAATAGCGTTGCTAATCACGTTTGCGGTTTCCATATGTGCCATTGTGCAGCGGAACCATGTCACC
ATCGGTCTTGTCATTCTCAACTATACCCTTCTTATTGATGCCATCGGCATTATTATTATTGGCACTTTTGTTTGG
TATTATACCCTTGGAGAACGTGCCAACTTCCATGTACTCTGGGCGGAAGCAAGCCGTGAAACTCGTATTACTCTC
CAGGACCAGCTCAAATGTTGTGGATATTTCAATGGAACAGATTTGGCTGAAGTCGGGGGTGATTTTTGTCAGACT
CAAGACTTTATCACCGGTCTTGCGGCCAACATCTCATCCAATTTTTGTGTCACGCCTATCACCGCCTTTGCTGAC
ATGACGTTGAACAACGCTTTCACCACTGTATATGGCTTTATGGCCATCGTATTGTGTTTGCTTTTGGCGTCGCTG
TGTGTTATCAAGAAGAGGCAAGAAGATGAACGTTTCAAGAGAATTGATGCCAAGCGGGGTGGAAGAGGTTTCGTT
TAA

>Lb_Pls1 manually annotated protein using EST
MVSRKLMLVWAALDFLLLVAGVVALVLSLTWKAPNLLMNMVLSSADLTAGTVLGIALLITFAVSICAIVQRNHVT
IGLVILNYTLLIDAIGIIIIGTFVWYYTLGERANFHVLWAEASRETRITLQDQLKCCGYFNGTDLAEVGGDFCQT
QDFITGLAANISSNFCVTPITAFADMTLNNAFTTVYGFMAIVLCLLLASLCVIKKRQEDERFKRIDAKRGGRGFV
*

>RO3G_14268_manually annotated gene
atgacgatttgctgtgcaag**gtttgtttcaaaactgcttatactttttttattgttattcattgcttttgatagac**
tgagtaaagcctatatgatcttaactaatgcactatttgcttcccttgggttagcattttagtatttggatcga
tgggcatgaagagcgattttatggctctagtttatttcctataaataccttcaagt**gtgagttttattatatat**
tttattttattttattttttactcagactaaaaaaggtttgagagaaaaagagtttaatttaaaaatgaaaatta
gggctagcaattcttggagctattgtttgtgtagcatccattttggcgccattggggcatttattagaaaaaaa
ttcattacttgtatttacatgattattatcttggttgctcttgttttacaagtcattattgggatcaaaatatat
aaggcctctgccaacatctttgcctatatgtctgatctttggacatcggctagtacaagttaccgtatagctctc
caaaatgag**gcaaatttgaatttttttatgtaatttaacaaaagaaagtaacgtggaaaatcttttctttgtag**tt
tagttgctgtggatttcaaacaagtatggataattatgctattaccgatcagtgccaaccaacagcttcttcaat
ccaagacgtccctccctgtgcagacatcctcataaactatgctaaatcagcatttggaaaggcttatttggttat
attcgcagctttaactttagaagtattggctatgacaaatgccatcactttattatgtacaagctttgacgctga
agatgaaaatgaaagacgtaaccataggaaatctggtataaggcttgacgagatgtcagtagagagtccaaccac
attagtagggtcttcttatactttgcctgaagaacaaaagaaacactatgctgattcccctattggtgattcata
tagctcttattctgcctacgatgttaattcgcccatacatgacagtaacagatatgaagtctacagccatcaaaa
tgattcaaatatttatcatcaggacaaccatggatttaacacaggcagccgtgttaacgcatactactag

>RO3G_14268_CDS_manual_annotation
atgacgatttgctgtgcaagtaaagcctatatgatcttaactaatgcactatttgcttcccttgggttagcattt
ttagtatttggatcgatgggcatgaagagcgattttatggctctagtttatttcctataaataccttcaagtgg
ctagcaattcttggagctattgtttgtgtagcatccattttggcgccattggggcatttattagaaaaaaattc
attacttgtatttacatgattattatcttggttgctcttgttttacaagtcattattgggatcaaaatatataag
gcctctgccaacatctttgcctatatgtctgatctttggacatcggctagtacaagttaccgtatagctctccaa
aatgagtttagttgctgtggatttcaaacaagtatggataattatgctattaccgatcagtgccaaccaacagct

tcttcaatccaagacgtccctccctgtgcagacatcctcataaactatgctaaatcagcatttggaaaggcttat
ttggttatattcgcagctttaactttagaagtattggctatgacaaatgccatcactttattatgtacaagcttt
gacgctgaagatgaaaatgaaagacgtaaccataggaaatctggtataaggcttgacgagatgtcagtagagagt
ccaaccacattagtagggtcttcttatactttgcctgaagaacaaaagaaacactatgctgattcccctattggt
gattcatatagctcttattctgcctacgatgttaattcgcccatacatgacagtaacagatatgaagtctacagc
catcaaaatgattcaaatatttatcatcaggacaaccatggatttaacacaggcagccgtgttaacgcatactac
tag

>RO3G_14268_ manually annotated protein
mticcaskaymiltnalfaslglaflvfgsmgmksdfygsslfpintfkwlailgaivcvasifgaigafirkkf
itciymiiilvalvlqviigikiykasanifaymsdlwtsastsyrialqnefsccgfqtsmdnyaitdqcqpta
ssiqdvppcadilinyaksafgkaylvifaaltlevlamtnaitllctsfdaedenerrnhrksgirldemsves
pttlvgssytlpeeqkkhyadspigdsyssysaydvnspihdsnryevyshqndsniyhqdnhgfntgsrvnayy

>RO3G_02583
MAACCARLSKVYMVITNLLFACLGLAFLAFGLIGLKTGFYGSSLFPTNIFKWLAILGAIVCVAAILGAIGAFVRK
NFITCIYMIIILAALALQVYIGIKFYKASANVSAYMSDLWTPASTDYRASLQNEFKCCGFQTNMDKYAKTDQCHP
TAKSIEAFPPCADILQSYAKSTFGKAYLVMFAALSLEVLAMANAITLLCTSFGGDEESERRKRRKSGIKLDDMSV
ETPTTLVGSSYNLYDEQKKYYAGSPGGGNASYSTQDVHSPVSNGYGMYNQNAYQSSHGNQYNAY

>Ss_Pls1_genomic_sequence
aaactttttttctgctttattcttttcatttctatttggttttcgtcttttctccgtcttttcttcatctctatc
tctcaatttgtgccaagcacaagttcaatcattggggycgatctctcgatacatttgtgcagggcattgattgct
tcatttttcattgtttctttttttggtcccttcattgcaataggccaatcagtcagtctatcatcacaaattta
gacgccggtcgtccttttcaccaggacagcttgctctttttttccttcatctacatttaattaaattcaattccga
ctttaagttgatatagctggtactattcaacaaaacacaaaATGGCCGACAAGATCCTTTGGACATACATTATCC
TAGATTTACTCTTTGTAGGTTCTGGAGCATTGTTACTTGGATTTGCCTTGAACACGAAGGCTGGTACTTCACAAG
CACTGAGTATTTCGAGTGTTGGGACGAAACTTCTATTGGAGGGTACTCCTTTGAATG**gttagtctaatacctcat**
atctcatatcctttcttgatgatatatgtgcaatgagctgacctaacttacccatcaaaagCTGCCATTGGCAAT
GCAATCTTAATCTTCATTTCCTTCCTCCTCTCCATTCCCGCCATGACCCTCAGCACCACCCGAGGCTGGCTCAAA
CTCCATGGCTACATGGTCGTCGTCTGCGCCATCTTCACCCTGGTCATAGGTCTCGATATTTGGTTTGGTACTCTT
CAATCCAAGGAATCTCTTCTCGATACATGGAATGCGCAAGGTCCCACCACGCAGAGTTTATTGCAGGAAGAATTT
GGATGCTGCGGTTATTTCAATAGTACGAGCGCTCCTGCATTCATTATAGATTCTACTTGTCCGAATGCTATTATT
GCAGCTACGATGCCGGGATGTAGTGGCGCTTTTGTTAAGTTCGATGGTTTATTTTTGGATGTTATTTTTACGGCG
GCTTTTGGTATTGTCG**gtatgttctctttctttttttcggaggtttgtttgaggtgctttgtatggtctagagtct**
agaggccagggttataagcgggggggagaagttaagggtgaatgggaaagccgaatggaattggttcactggaaaa
attgggataagagctaacgaaaataatgtatagGTCTCGATGTAGCAGTTATCTTCGGTATTGCGATGCTTAACA
AAGATCGTAAGGAGAGGGAAAGATATAGGTTTATTGATGAGAAGAACGGGATGGGATCTTTTTAGggtggtgact
caaggttg

>Ss_Pls1_cDNA sequence
ATGGCCGACAAGATCCTTTGGACATACATTATCCTAGATTTACTCTTTGTAGGTTCTGGAGCATTGTTACTTGGA
TTTGCCTTGAACACGAAGGCTGGTACTTCACAAGCACTGAGTATTTCGAGTGTTGGGACGAAACTTCTATTGGAG
GGTACTCCTTTGAATGCTGCCATTGGCAATGCAATCTTAATCTTCATTTCCTTCCTCCTCTCCATTCCCGCCATG
ACCCTCAGCACCACCCGAGGCTGGCTCAAACTCCATGGCTACATGGTCGTCGTCTGCGCCATCTTCACCCTGGTC
ATAGGTCTCGATATTTGGTTTGGTACTCTTCAATCCAAGGAATCTCTTCTCGATACATGGAATGCGCAAGGTCCC
ACCACGCAGAGTTTATTGCAGGAAGAATTTGGATGCTGCGGTTATTTCAATAGTACGAGCGCTCCTGCATTCATT
ATAGATTCTACTTGTCCGAATGCTATTATTGCAGCTACGATGCCGGGATGTAGTGGCGCTTTTGTTAAGTTCGAT
GGTTTATTTTTGGATGTTATTTTTACGGCGGCTTTTGGTATTGTCGGTCTCGATGTAGCAGTTATCTTCGGTATT
GCGATGCTTAACAAAGATCGTAAGGAGAGGGAAAGATATAGGTTTATTGATGAGAAGAACGGGATGGGATCTTTT
TAG

>Ss_Pls1_Protein 225aa_using_cDNA
MADKILWTYIILDLLFVGSGALLLGFALNTKAGTSQALSISSVGTKLLLEGTPLNAAIGNAILIFISFLLSIPAM
TLSTTRGWLKLHGYMVVVCAIFTLVIGLDIWFGTLQSKESLLDTWNAQGPTTQSLLQEEFGCCGYFNSTSAPAFI
IDSTCPNAIIAATMPGCSGAFVKFDGLFLDVIFTAAFGIVGLDVAVIFGIAMLNKDRKERERYRFIDEKNGMGSF

>Bc_Pls1
MADKILLTYVILDILFVGSGALLLGFALTTKTGTSQAPTIASVATDLLLMGTPLNAAIGNAILIFFAFLISIPAM
LLSTTRGWLKLHGFFVVVCGLFTLVIGLDIWFGTLESKQSLLDTWIAQSATTQSLLQEQLSCCGYFNSTSAPAFV
IDSTCPNAIIAATMPGCSAAFVKLDGLFLDVIFTAAFGIVGLDVALIFGIAMLNKDRKERERYRFIDEKNGTGSF

>Cp_Pls1_genomic
ATGCGGGACAAACTGCTCCTCACTTACCTGGTTGCAGACGTCCTCTTTTTGGGAGGCGGCGCCTTAATTCTCACC
GTTGCCTTGACGGCCAGAGATAAGATTCGATCTGCGCCGACGCTGGATAATGTCGCAGAGAGATTGCTGCTTGCT
CACTGTCCCCAGCTAG**GTGCGGCGATCCAAACTAGCACATTTTGCAGTGTGGTCTTCTTTACTTCCCAAGTGGTT**
GCTGACGGTTCTATTTACATTTACAGGAGAGATTATCAATGCCGGATTCGTCTTCTTCACGTTCCTCCTTTCGAT
CCCAGCCATTATTCAATCAAATGATCGGATATGGATGAAAATCCACGGCTGGATGGTTGTGATAAGCGGATTCAT
CACACTGATCATAGGATTGATCATCTGGTTTTTGACGTTAAGGACAAGGTCAACATTGAGCGATGCGTGGGGAAA
TGAGACACCAGAGGTGCAGAGCTTATTGCAACAACGA**GTATGTCGATCGTTCTTCCTCAGAAATACACCTTTCTT**
GCTGGTTTTTCTTCTTCGAGAACTAAAGAGAGGTAAATTAGTTTAATTGTTGCGGCTACTTGAACAGCACCTCGC
CGCCATTTCAGGTCGATAAAACGTGTCCGACCGATCTCGATGCGGCACAAAAACCAGGATGTGTTGGCCCGTTTT
CCAATTTTGCGAACAGTTTTCTGGATGTGATTTTCACTGCGGACTTTGGCATAGTTG**GTATGAAGACTTCCATGT**
CGCAGGATGATGTGTTTCAGAGATCTGTGGATGCTGATTGTTCTCGTCTTTTAGCTATCGATGCTCTTCTACTTC
TCTGTGTTGCAATGGTCCTCAAAGACCGGAAGGAGCGCGCAAGATATCGGTTGATTGACTCCAAGAACGGGTTCG
GTACAATATAA

>Cp_Pls1_cDNA
AGCTGTAAAATAGTACAGCCAGCCCGGCACTCTCGTTCATTGGCGCTTGCAGATTTTAATTTAATTCCTTTATTT
TGTTTGCAAGTCTATCGACGCTGGTCGTCAGTTGCTTTCGTGTCCATAGCTTGTTCGCTTCTTGCCAGACGAGAG
AGGGAGCAGACCAAAAGGTCATCTGGCGTGACATACCGCTGAATTGATAAAAGATGCGGGACAAACTGCTCCTCA
CTTACCTGGTTGCAGACGTCCTCTTTTTGGGAGGCGGCGCCTTAATTCTCACCGTTGCCTTGACGGCCAGAGATA
AGATTCGATCTGCGCCGACGCTGGATAATGTCGCAGAGAGATTGCTGCTTGCTCACTGTCCCCAGCTAGGAGAGA
TTATCAATGCCGGATTCGTCTTCTTCACGTTCCTCCTTTCGATCCCAGCCATTATTCAATCAAATGATCGGATAT
GGATGAAAATCCACGGCTGGATGGTTGTGATAAGCGGATTCATCACACTGATCATAGGATTGATCATCTGGTTTT
TGACGTTAAGGACAAGGTCAACATTGAGCGATGCGTGGGGAAATGAGACACCAGAGGTGCAGAGCTTATTGCAAC
AACGATTTAATTGTTGCGGCTACTTGAACAGCACCTCGCCGCCATTTCAGGTCGATAAAACGTGTCCGACCGATC
TCGATGCGGCACAAAAACCAGGATGTGTTGGCCCGTTTTCCAATTTTGCGAACAGTTTTCTGGATGTGATTTTCA
CTGCGGACTTTGGCATAGTTGCTATCGATGCTCTTCTACTTCTCTGTGTTGCAATGGTCCTCAAAGACCGGAAGG
AGCGCGCAAGATATCGGTTGATTGACTCCAAGAACGGGTTCGGTACAATATAAACCGGTGAGGCGGTTGATTCCA
AATATTTTTCTTCGGCTTACGACAACTTCACTTCATCCAACGGTTTCTGATATGTCAGTGATAT

>Cp_Pls1_using_cDNA
MRDKLLLTYLVADVLFLGGGALILTVALTARDKIRSAPTLDNVAERLLLAHCPQLGEIINAGFVFFTFLLSIPAI
IQSNDRIWMKIHGWMVVISGFITLIIGLIIWFLTLRTRSTLSDAWGNETPEVQSLLQQRFNCCGYLNSTSPPFQV
DKTCPTDLDAAQKPGCVGPFSNFANSFLDVIFTADFGIVGAIDALLLLCVAMVLKDRKERARYRLIDSKNGFGTI

>Lm_Pls1_genomic
tgaaggagtagaaagaccgtagctcttgcccgcctttcctttgctttcccttcttccctttycttcttctcttct
ctcctaatcgtttgcctttacgcgttactcgacgcagtctttcgagcccgccggacggctcctgcgttcgacttc
acttcttcgcttcccatctactccgttttcttcttctttcagtccaatccagtccagtccagtcaagtctcttgc
gtgcacttgcttctataacacagcaataccgccaatATGCCCACCAAACTGATGATGGTCTTTGTCGTCATGGAC
CTCATCTTCGCCGGATGTGGAGGATTGTTACTGGCCTTCTCTCTGATATCTGAGCAGACGATGCGGGACTCGCCG
ACGCTCGACAATGTCACACAACATATACTGCTGGGCCAGTGTCCGTTGACAG**gtacgacgttttccaaacgggtc**
gagggaagaaagagttgggattcttcaatagcatggcaccactgtggcgcaagccccgtcctactcgactctaga
ccacttgggaaacttcaatagatgcaaactgacaggtggttagCTGGCGTCGTCAACGCAATCTTCGTCTTCGTC
ACCTTTTTGCTGTCCCTCCCCGCCCTCTTCCTACCCACTAACAGAGGCTGGCTCCGCGCCCAAGGCTGGCTGGTC
GTCTTCTGCGCCACCTTTACGCTTGGACTTGGACTGACCGTTTGGGTGGAAACGCTGCAAATCCGTCGAAATCTG
AGCCTGCTTTGGGGTCGCGAGACGCCCCTGATTCAGAGCCTGCTACAACAAAAG**gtttgtccattcttctcgacg**
tggaaggatgcctcgccgtccatggtactgacaagagatgcctacagTTCGACTGTTGCGGCTATGTCAACTCGA
CCACGCCTCCATTTGTCCAGGACAGCACATGCCTCAACACCCTAGTCGCCGCGCAAAAAGGGGGCTGCATCGCCA
AGTTCTCGTCCTTTGCCAACAGCTACCTCGACCAAATCTTCACGGCTGCCTTTGGCATTGTCGGCGTCGACTTCA
TCTTGGTGCTCTGCGTGGCCATGGTGCTCAAGTACAGATTGGAGCAGGAACGGTACAGACATATTGATCAGAAGA
ACGGTCTGGGTGGGATTTAGgtgttgggaagatgataaaaaaatgaaagaagaggaggaagaaggattagcgttg
cgggagaggatgacggggatgaaacatacttgcacatgcaagtatcaaatcacaatctcggaccctgctttcttt
ccattgggggtgaggtttcggtcttggcatggagatggcaattgtgatgtgaattgatggtaccgagtaatgata
tggcaagagacactggctgccctgtgaaatgacagtacctagaaatgacatgggcaagaggcacctggctgccct
ggagataaccaaaaaaaaaa

>Lm_Pls1_genomic_cDNA coding region
ATGCCCACCAAACTGATGATGGTCTTTGTCGTCATGGACCTCATCTTCGCCGGATGTGGAGGATTGTTACTGGCC
TTCTCTCTGATATCTGAGCAGACGATGCGGGACTCGCCGACGCTCGACAATGTCACACAACATATACTGCTGGGC
CAGTGTCCGTTGACAGCTGGCGTCGTCAACGCAATCTTCGTCTTCGTCACCTTTTTGCTGTCCCTCCCCGCCCTC

TTCCTACCCACTAACAGAGGCTGGCTCCGCGCCCAAGGCTGGCTGGTCGTCTTCTGCGCCACCTTTACGCTTGGA
CTTGGACTGACCGTTTGGGTGGAAACGCTGCAAATCCGTCGAAATCTGAGCCTGCTTTGGGGTCGCGAGACGCCC
CTGATTCAGAGCCTGCTACAACAAAAGTTCGACTGTTGCGGCTATGTCAACTCGACCACGCCTCCATTTGTCCAG
GACAGCACATGCCTCAACACCCTAGTCGCCGCGCAAAAAGGGGGCTGCATCGCCAAGTTCTCGTCCTTTGCCAAC
AGCTACCTCGACCAAATCTTCACGGCTGCCTTTGGCATTGTCGGCGTCGACTTCATCTTGGTGCTCTGCGTGGCC
ATGGTGCTCAAGTACAGATTGGAGCAGGAACGGTACAGACATATTGATCAGAAGAACGGTCTGGGTGGGATTTAG

>Lm_Pls1_using_cDNA
MPTKLMMVFVVMDLIFAGCGGLLLAFSLISEQTMRDSPTLDNVTQHILLGQCPLTAGVVNAIFVFVTFLLSLPAL
FLPTNRGWLRAQGWLVVFCATFTLGLGLTVWVETLQIRRNLSLLWGRETPLIQSLLQQKFDCCGYVNSTTPPFVQ
DSTCLNTLVAAQKGGCIAKFSSFANSYLDQIFTAAFGIVGVDFILVLCVAMVLKYRLEQERYRHIDQKNGLGGI

>Nc_Pls1
MSKVLLAYVVADGLFLLMGIFMIAFSVIVQNIQFEVPTEGQQAARNLLYQRFPLTAGIVNAVFIFVTFLFTIPGI
ITPARGWLKLGGWMTTVCGIFSLIIGLYLWIMTLKTKADFAPFYFSQPPEIQELMQSAFKCCGYFNSTSPAFITD
DICSSPAAAALMRPCATPITSFANVLIDNIFTAVFGMVGIDVVLVMATACLLKERKERERFRHIDEKSGAIGF

>Pa_Pls1
MVNKVQAAYVVADGLFLLMGIFIIGFSVIVGNIRDEIPENGRQAARNLLYQRFPLTAGIVNAVFIFLTFMVTLPG
LATQSRGWLKLGAYMTTFCGLFSMILGLFLWILTLKTREDFAPLYWAQPANVQQLMQAEFNCCGYFNSTSPAFVT
DATCSSPAAAALVRGCATPITSFANIFLDNIFTAMFGMVGIDFVFVMATACLLKDRKERERFRHIDEKSGYGRI

>Cg_Pls1_manually annotated gene
atggcgaacaagattcagatcgccttactttccgccgatggcctctttttgctcatgggcgtatttcatccctc
ggcttctccgtcattgtcaacaacatcaaggatgaggtaccagaaaacgggagacaagcggcaaggaacctcttg
taccaggagtttccgctgacag**gttggtggttgccgcatcagttcgcggatgggaacctttggctaactagcata**
cagccggtattgtcaacgccatcttcgttttcatcaccttcttcgtcacgcttcccggcctcgccacgtcctctc
gcaaatggctcaagctcgcgagctacatggcggtggtctgcctgatattcagcatgattctcggcctgtacctat
ggatccagaccctcaagctgagggaggacttcgcccctcgtttcaccgcccaacccaacgacatcaagagcctca
tgcaggtcgag**gttagtatctcaatcaccacaaatcccctcttccaaagccgggccccggccccttcccgaataa**
ccccccactaacactccgcttacagttcaactgctgcggctatttcaacagcagcagcccggccttcgtgacgga
cgcgacgtgcgcgagcaaggccgccgcggccgtcatgcgcgggtgcgcgaccccgatctcgggcttcgccaacgt
cttcattgacaacatcttcacggcgctcttcggcatcgtcggcttcgacgtcatgtttattatggccaccgcctg
cctgctcaaggatcgcaaggagcgcgagcggttccagcatattgatgagaagaatgggtttgggaggatctaa

>Cg_Pls1_CDS_manual_annotation
atggcgaacaagattcagatcgccttactttccgccgatggcctctttttgctcatgggcgtatttcatccctc
ggcttctccgtcattgtcaacaacatcaaggatgaggtaccagaaaacgggagacaagcggcaaggaacctcttg
taccaggagtttccgctgacagccggtattgtcaacgccatcttcgttttcatcaccttcttcgtcacgcttccc
ggcctcgccacgtcctctcgcaaatggctcaagctcgcgagctacatggcggtggtctgcctgatattcagcatg
attctcggcctgtacctatggatccagaccctcaagctgagggaggacttcgcccctcgtttcaccgcccaaccc
aacgacatcaagagcctcatgcaggtcgagttcaactgctgcggctatttcaacagcagcagcccggccttcgtg
acggacgcgacgtgcgcgagcaaggccgccgcggccgtcatgcgcgggtgcgcgaccccgatctcgggcttcgcc
aacgtcttcattgacaacatcttcacggcgctcttcggcatcgtcggcttcgacgtcatgtttattatggccacc
gcctgcctgctcaaggatcgcaaggagcgcgagcggttccagcatattgatgagaagaatgggtttgggaggatc
taa

>Cg_Pls1_ manually annotated protein
MANKIQIALLSADGLFFAHGRISSLGFSVIVNNIKDEVPENGRQAARNLLYQEFPLTAGIVNAIFVFITFFVTLP
GLATSSRKWLKLASYMAVVCLIFSMILGLYLWIQTLKLREDFAPRFTAQPNDIKSLMQVEFNCCGYFNSSSPAFV
TDATCASKAAAAVMRGCATPISGFANVFIDNIFTALFGIVGFDVMFIMATACLLKDRKERERFQHIDEKNGFGRI

>Mg_Pls1
MANKILVAYIFADFLFVLMGALMLGFSIVVGNVRDEVPTEGNQAARNLLYQKFPLTAGIVNAIFIFITFLLTIPA
LSTPARGWLKMSGYLVVVNALFSLVIGLFLWIMTLKTRDDLFPIWVQQTPQVQSLMEVSFKCCGYYNSTAPAFVT
NQVCPSPAASALMRGCATPITSFANVFVDNIFTGVFGMCGIDGLLVIATACLLKDRKEQERFRHIDQKTGPMSTL

>Cl_Pls1
MANKVLMAFVAADVLFAITGALILGYSLINQNTMNEVPTEGVQAAIRLSTKQFPLTAGVVNAVFIFITFLFTIPG
MITPARGWLKVSGYMVTFCGVFSLIIGVYLWVLSLTTKADFAKLWISADPSVQELMQPAFQCCGYFNSTSPAFIT
DVQCPSPAAAALQRGCAAPVTSFVNVFLDNVFTAVFGMVGVDALLVVCTAMLLKDRKERERYRHIDEKAGYRGF

>Gz_Pls1
MVDKIFLTTVCADILFLGSGVMELVFSLVVRSQMNDMATDGESATRNLLYQRFPLTAGIVNAIFILVTFAATLPG
LVMPARSFLKVSGYMVTVCSIFTMCVAVFLWVMTLRMKEQFFNIYIEQDPDVQSLIQNSFQCCGYNNSTSPAFVM
DSTCTSPASSALLRGCATAISSFANLHIDGIFTVLFGLVGIDAIFVLCIACLLKDRKERERYRHIDEKSGYRQI

>Tr_Pls1
mpdkvfvatlvadalflasgamelgfsiaalnlkdkaptdghdatrhiiyqhfpltagianailvlatflftipa
lalakrsllkisgyfitlcavftlavglylwimtlrlketfepfyaaqdttvqsmmqtsfqccgylnstspafit
detcpspaaaallrgcstnvasfanafindlftavfgmvgidallilaiaclvkerkemeryyiidqkrdygrl

>Sn_Pls1
MPTKLMMVFVGFDFLFAGCGGLLLGFSLMSEQSIRNTPSVDNVTQNLLLGQCPLTAGVVNAIFVFVTFLLSLPGL
FLPTNRGWLRAQGWLVIICATFTLGLGLAIWLETLQTRKNLSNIWGRERPLIQSLLQQKFDCCGYTNSTTPPFVQ
DATCTNPLVAAQKGGCIGKFSSFANRFLDQIFTAAFGIVGIDVILVLCTAMVLKYRMEQERYRHIDEKNG

>Cc_Pls1
mvsrklmaayaffdvcllaagivalvlsitwrapdvlmnmvlsnseltagtilgvsllvtfvisvaavvqrshvt
lgfvilnwallldalgivvigtfvwywtlqpranfrvlweaaspatriilqdrlkccgyfngtdlaeiggsfcts
revidalpvdpefmtnfcvtpitafadstlnnifttvygymaiviclfltslcvikkrqederfkridakrggrg
fv

>Pc_Pls1
mvssrimafygfvdlwllaagllsvimsfvwrapnlmlnftisnsdltaglvlgimllftffisiiaiaqrnhvt
sglvflnwtligdaiavlvigtfiwfyslqqrnnyyevfkvqtadtrraiqdkfsccgymtpnetftvlggfcan
qtfvdslfnksapdqnacvgpitaftdftlnnifttiygfmaiiislflaslcvihqrneserfqrieakrggkg
fv

>Nh_Pls1
MANKVFLAAVAADILFLASGILELVFSLVVRSQMNDAPDDGERATRNLLHQRFPLTAGIVNAAFILATFAVTLPG
LVMPARSILKISGYMVTFCAVFTMCVGVFLWVMTLKMREQFFDIYIAQEPEIQSLIQTSFECCGYYNSTTPAFVM
DTTCTSPASAALLRGCATAISSFSNLHIDSIFTVVFGIVGVDAIFVLCIACLLKDRKERERYRHIDEKSGYRQI

>Fv_Pls1
MVDKIFLSTVVADALFLGSGVMELVFSLVVQSQMKNTPTDGEDVTRNLLYQRFPLTAGIVNAIFILVTFAVTLPG
LVMPARSFLKVSGYMVTVCAIFTMCVAVFLWVMTLRMSEQFFNIYIDQEPDVQSLIQDSFQCCGYFNSTTPAFVM
DSTCSSPASAALLRGCAASISSFANLHIDSIFTVLFGIVGIDAIFILCIACLLKDRKERERYRHIDEKAGYRQI

>Tsp2A_Cc
MAPSPFSIQDSPRSSRRASVAGSAYSAAGSTRHMLADYNPDRNSASFLDVPRYNGASPGPSTPSTPLSPVGSATG
LSLMVNYIPSKFGSTLSSRKAYRNGAGQGPAVPKQGGGLAAFKSNENRIGGKDLRWTKFKWVLFFSNLLLTLYSL
GAMIIILLTWFNVFEKSEIIRFGNHTELALSTTAAVLGLVTSLIGWAGILLNNRAFLAVYCLLTWITFIFIVAPG
YLTFRKQNYSLEGKVNEQWSRRLGPVARMRIQSQLHCCGYFSPYVEATVTQTCYSRSVLPGCKKPYLDYQRHILG
VFWKIAFSIVPAHLLVMIAALLCSNHVTYRFGKGMMPEAYRLNPDSMAVIMDQYAAQLAEQYGPEVANKVLEKSR
STMSLSTAYNSSPR*

>Tsp2B_Cc
MDNPFSAVYDSPSFALPAGSAFLLLRHLAPVSATSTRPMLDLKDFDQSFSSDTALVGGDVKDTKDTTATGRDEKS
RPSSFLAGFGGSIQLPRNSSYGANLSSGAQTPTSTTGLSLSVNYLPSKFSGALLSRRSSRVDLGGAGGGGDGKEV
VDVPKSGGGTAAFKSNEQRIGGASGQKLRWTRFKWILFFTNLLLSLYSLGTFIVCLLTWFDVWKHAEVIRFGNRT
ELVLSTLASSLGILTSVIGWAGILLNNRMFLSIYTLMTWITFIFLVTPGYITYRKRTYNLEGKVNQQWSRNLGPI
ARTRIQNQLSCCGYFSPYVEATVTQTCYSRSVLPGCKLPYLSYQRRILQVFWKTAFAVVPAHVVVMVAALLCSNH
VTYRFGKGMMPEAYRLNMDSMAVIMDQYAAQLADQYGPEVAHKVLERSRSNMNLLQKQ*

>Tsp2C_Cc
MLGASTGGLSSGDASNVSLSVNYLPTKFSSGMLNAELEGKSGTRRRRGKGAIRDVDVMLKVPKMGGGLDAFKAGE
ARMGGEDEDADLYDTSRSTSSSFGKVMFWKKDKKRRQGSSPPKEMRWNRFKWILFISNTVLTIYSFACLIVCLLL
WFGAWQHADVVRVGNRTELIISTLASCMGLFTCLLGWAGILMNNRVFLAIYSFLLWITFIFLVIPGYLTYKRRTF
NLEGKINAQWSRDLGPVGRITIQNSLSCCGYFSPYVEATVSQTCYSRSVLPGCKKAYIDFERDVLQKWYTVVFAI
VPVHLVIMTAALLCSNHVTYRFGKGMMPKRYRLSLSSMALIMENYAQQLVERYGTDVASDIMSQSKSQVKLGSQL
PQDMAPTLPYLALGNVGSLSSAGTSLLNNSSK

>Pc_Tsp2
fasvifilvpilppgwtacgrptwtirairaralvpshsrtssvnshspslqsaaastrymlagmsspnleppsa
pfagrrestrsfssgnspllkdsdklstslsvnylpakfsntvvaqrrrngkgaggymmpkqgggreafrsnear
mpgagdedydgvdvfgnnkeggrtkpkarwnrfkwclflanilfsayslagliaclliwfnvwthadiirvgntt
eliisttaaslavftsligwsgiilnnraflawytfllwicfallvtpgyitykkrtfnlegkinaqwsrslgia
grlrvqnqlrccgyfnpfveatvsqtcyarsvlpgckgpylkfertvlerwytvifsvvpaqllvmitallcsnh
vtyrfgkgmmpkayrlnmnsmavimdnyanqlaeqygndvaseilarsrsnlqldamptmpysnnnsqqssyvgk
ydqipgkapeg

>Tsp2_Cn
MPSDTHRRSRFGNFASKFAPHSDPQRRSSHSGSLLSPLDPNSPQSGSKENYYGRPSSPTYSDQLSSNNHGQPQEV
SYRIDDDLVPPVAPFAPQADSALSSRRSSISFLADKDSLKNRASSLSLNYVPAKFTRLHAPGDYAHRRKQGGGRD
AFASNAQRMGQVGTVDDDEGVIFQISESGLKRKKPKLRWNRFKWVLFLANSVLFIYGMATLVCAILVWLNIFYQS
DVIRVGNRTELIISTVAASMITFTSLLGFAGIFLNNRTFLAIFTLLLWVDFGLLVAPGYITYKQKTFNLEGKINS
QWSRYLGTEGRLRIQDALRCCGYYSPFVEATVSPLCYSRSNFPGCKSQYLRLERRVLGIWFTVSFAIVPAHLLII
LAALLCSNHVTYRFGKGLMPERYRLDLGSMAVIMDEYAGQIAAQYGPTVAQEAVERSSINLATPTPCRSEYDVSL
LSVPNSRRGSSSNLEAMRRGALPGSTRGVSLYDPSNPRASMDHRPESSFGGNTHIASGSGSHNGSHQADESVASF
SNDDHRRR

>LbTsp2_A
MPRQPSLRVSSSTLSTVRRRVSAQEPPHEEDRGPHRKPTIKLLPKTQESLPQSFSSSFLNLPYEITSPETYAPST
FPDPESLVVPEPESPVLTQEQLSNIPLTPRPDPPSRASTACSWESTETRVSQISRLPTPDFTNPSQSTLQRGAGS
LRMLLPKRLSYISFNIPQTAFWSGGRPELKAQSEARHIPPQIPFWAGIRPNLNALRNQSEGSAKTRITARTTNSV
DSGHHALIPSIGTTDKFTHKWPKPQSLKYLNSRTNSTSSNGSSVHLDQAASLALEEGQGLGVGSVMRWTTFKWCL
VLSVSTVFVYGAAGLACAIMTWFRTWDKADVMSVADGDILILITLAASILLFTSLVGMSGTLLNSRPILAVYTLL
LWPALASLLAVGYISYHRATFSLDHKLNLSWSQYYTPLGRLLIQNSLHCCGFYSALHEATFSKRCYPRTSLPGCK
GKLYRFDRENLAAIWMAVFSLVPLHLINILVALLCANHVTRTFGKGITPKRYRLSSEDVKADAERILSGIDGRGT
VRPVVQPEYSRAGSSGVFREDREDRTTLLKDEDYWYTL

>LbTsp2_B
MENNSPRSSSRLSIAASAYSAAGSTRHILGGHNNVSSGDYDPPSSPPGINDSRDTLSHYRGPTGTTATGVSLMVN
YLPSKFSASLVSRKGDKGVEPHLPKNGGGVEAFRSGESRMSGRRLRWTKFKWILFCTNFCLSLYSLVSLVICLLT
WFDVWAHADVVRVGNRPELIISTIAASIGIFTSLMGWAGILMNNRGFLAMYTFLTWITFAFLVTPGYMTYRHRTF
NLEGKINAEWSRKLGASGRLRIQDQLECCGYFSPFIEATVSSTCYARALLPGCKLPYLTFQRLVLGRWYKAAFSL
VPLHIVVMIAGLLCSNHVTYRFGKGMMPEAYRLNASSMAVIMENYASQLAEQYGSEVADEVYKLSRSNLSLVGDM
PSTPINTARRPAQAPYHAKYESLGGGH

>LbTsp2_C
MPSRNPPIDLPNLSYSYNAPGSPSSRNDSFGPDSKGDMSMLSKGDMSTASSSVSLSVNYLPSKFSNSMLAYPKNR
KSKKGINPGYPKMGGGVEAFRTGEARMGGENEDEDGVPTRKSWFGARPNKKLRWNRFKWVLFTANTLLTLYSLCS
LIICLLIWFDVWSHADVVRVGNRPELVVSTLAASMGIFTATLGWAGIMMNNRSFLAVYTFLLWIAFIFLLVPGYL
TYKRHTFNLEGKINAQWSRDFGADGRLRIQNQLSCCGYFSPFVEATVSQTCYARSMLQGCKSDYLAFERRVLRKW
YTTVFSIVPVHIVIIFAGLLCSNHVTYRFGKGMMPKAYRLSMTSMAVIMDNYATQLAEQYGSEVASDIINRSRSN
LHLDSMPTMPYTPRNKTQYNDTTKYDALNKTLVDDSI

>LbTsp2_D _manually annotated gene
atgccaaccactggtcagaactcgacgacgggtctctctttaaccattaactatgtcccatccaagttttctggg
accattctcaggcgcaaaacgactgccaagatgccaaaacttggtgggggcgtcgaagcattccggagtggtgaa
tcccggatgcctggacaaacctttggcaacgaaaatagaatttccatgttcggagggaaattgcaagtagcgaac
aagtacaggtggaacaagttcaaatgggtgctcttttctccaacctctgt**gtacggccttcattcattattcaa**
gtttttcttaactatcactcattcccacatctactacagctgactacctatgcccttgtctcccttgttgcctgt
ctactgacttggtttgacgtgtggcaacacgccgacgtgatgcgtgttggtaatcgtcctgaactcatcgtatcc
accattggtgccaccatcggtgttctgacatctatcataggatgggctggtatcctccttaacaatcgctcgttt
ctcgctgtttacaccttcatgacttggattaccttcgcattcctcattactccgggctatatcacgttccgccgg
agcgaatacaacctcgagggtaaaattaactctcagtggtcaaaaacacttggtgccgagggccgacttaggatt
cagaatcagcttgaatgctgtggttattttagcccatttgtagaggccactgtcagccagacttgttacgctagg
agcttgttacctgggtgtaaattgcgatatctaacattcgaacgcaatattatgaggaagtggtatcacgcttcg
ctcgggctggcaccattgcatatccttgtcatgattgcggtcctgttgtgctcgaatcacgtcacatatcgcttt
ggaaagggcatgatgcctgaagcataccgtttggacttgaatagcatggccgttatcatggaaaactatgcaaa**g**
tacgccttcctagttctctccacgtcgaaatacttcctgacttctcctttgttcagtcagttggcggaagaatat
ggagcagaagctgcttcagagattctcaagcgatctcgatcaaacctcaatctcagcgagataccttccaatcca

tttgagaaccctccacctacaccaaatcttgacgattcaaaataccaatcagctactaagaaggggcgagaaccc
ttctag

>LbTsp2_D_CDS_manual_annotation
atgccaaccactggtcagaactcgacgacgggtctctctttaaccattaactatgtcccatccaagttttctggg
accattctcaggcgcaaaacgactgccaagatgccaaaacttggtgggggcgtcgaagcattccggagtggtgaa
tcccggatgcctggacaaacctttggcaacgaaaatagaatttccatgttcggagggaaattgcaagtagcgaac
aagtacaggtggaacaagttcaaatgggtgctctttttctccaacctctgtctgactacctatgcccttgtctcc
cttgttgcctgtctactgacttggtttgacgtgtggcaacacgccgacgtgatgcgtgttggtaatcgtcctgaa
ctcatcgtatccaccattggtgccaccatcggtgttctgacatctatcataggatgggctggtatcctccttaac
aatcgctcgtttctcgctgtttacaccttcatgacttggattaccttcgcattcctcattactccgggctatatc
acgttccgccggagcgaatacaacctcgagggtaaaattaactctcagtggtcaaaaacacttggtgccgagggc
cgacttaggattcagaatcagcttgaatgctgtggttattttagcccatttgtagaggccactgtcagccagact
tgttacgctaggagcttgttacctgggtgtaaattgcgatatctaacattcgaacgcaatattatgaggaagtgg
tatcacgcttcgctcgggctggcaccattgcatatccttgtcatgattgcggtcctgttgtgctcgaatcacgtc
acatatcgctttggaaagggcatgatgcctgaagcataccgtttggacttgaatagcatggccgttatcatggaa
aactatgcaaatcagttggcggaagaatatggagcagaagctgcttcagagattctcaagcgatctcgatcaaac
ctcaatctcagcgagataccttccaatccatttgagaaccctccacctacaccaaatcttgacgattcaaaatac
caatcagctactaagaaggggcgagaacccttctag

>LbTsp2_D manually annotated protein
mpttgqnsttglsltinyvpskfsgtilrrkttakmpklgggveafrsgesrmpgqtfgnenrismfggklqvan
kyrwnkfkwvlffsnlclttyalvslvaclltwfdvwqhadvmrvgnrpelivstigatigvltsiigwagilln
nrsflavytfmtwitfaflitpgyitfrrseynlegkinsqwsktlgaegrlriqnqleccgyfspfveatvsqt
cyarsllpgcklryltfernimrkwyhaslglaplhilvmiavllcsnhvtyrfgkgmmpeayrldlnsmavime
nyanqlaeeygaeaaseilkrsrsnlnlseipsnpfenppptpnlddskyqsatkkgrepf

>RO3G_17009_ manually annotated gene
ttattctgttatggacttggcacattattattagctcttttaacttattttaaat**gtaaggagcatcagacatca**
agatctttttttgattcactctccccttttttatatacagtctatttgcgtgcagatgttgttctggttggagaaa
aaacgattctggatt**gtaaggagagagagaaagaaagagagagagaatgaatgataatgaacatgtattctttag**
tggtccttgctacaggtgtcatttgcttattgacatcccttgttggttacacgggcatcatgttgaataatagag
ccattttaactgtttataatctattgctttggccttgttttggtatcattgctgctatcggttatacagcctatc
gtaaaaacaagtggaatctggaaggaaagctatcttatcagtggcattataaactgaattcggatggtagagcac
gtatccaagccaat**gtaagtccctctccttcaatatccacagtgactgatctgaagatag**cttcactgttgtggt
tacaagtcattttcagattaccacgaaagatccaatcaatgcttccctcgtaccttattacctggctgtaaattc
aaatatcagacattcacaaaagaagcactgacaatcacttggattgtggccttctcaatgatccctctccatctc
tttgtcttgttctctggattgttatgttctaatcatatcaatcgtaaatttggtaaaggacttccacctaaaatc
tatcgtttggactatcaaggcatcgttgctggtacacctactggaagctcacttaatttacacacagatggttta
cagcaaagacatgttcaatcataa

>RO3G_17009_CDS_manual_annotation
ttattctgttatggacttggcacattattattagctcttttaacttattttaaattctatttgcgtgcagatgtt
gttctggttggagaaaaaacgattctggattgtgtcatttgcttattgacatcccttgttggttacacgggcatc
atgttgaataatagagccattttaactgtttataatctattgctttggccttgttttggtatcattgctgctatc
ggttatacagcctatcgtaaaaacaagtggaatctggaaggaaagctatcttatcagtggcattataaactgaat
tcggatggtagagcacgtatccaagccaatcttcactgttgtggttacaagtcattttcagattaccacgaaaga
tccaatcaatgcttccctcgtaccttattacctggctgtaaattcaaatatcagacattcacaaaagaagcactg
acaatcacttggattgtggccttctcaatgatccctctccatctctttgtcttgttctctggattgttatgttct
aatcatatcaatcgtaaatttggtaaaggacttccacctaaaatctatcgtttggactatcaaggcatcgttgct
ggtacacctactggaagctcacttaatttacacacagatggtttacagcaaagacatgttcaatcataa

>RO3G_17009 manually annotated protein
lfcyglgtlllalltyfkfylradvvlvgektildcviclltslvgytgimlnnrailtvynlllwpcfgiiaai
gytayrknkwnlegklsyqwhyklnsdgrariqanlhccgyksfsdyhersnqcfprtllpgckfkyqtftkeal
titwivafsmiplhlfvlfsgllcsnhinrkfgkglppkiyrldyqgivagtptgsslnlhtdglqqrhvqs

>RO3G_08988_genomic_correction of RO3G_08988 using EST
atgaacccaacactcacagcagcagccaggggacatccttatgtgtctcaagccagttcatccgccagcctacag
gacatcaggcctaatccaaggccttatgattcctatgaatcatttcctatcaatggcttacaagagcctgtcttg
ccttggatgaaatcagaatcgaggcagtcattaggatctcgtagttcgcttgatacaaccagtcttcatatcatg

aaaggtcaattccccgatccttcagctaaaaaaaaacaaaggtggtcaaagcataaatggtggttactcttatca
aatactttg**gtaaagaaaaaaattttaagaaaaaaaaaaaaagagctgattgtttttttttaaag**ttattttgtt
atggattagttatattattattagcactttaactttttttaaat**gtaaaaaaagaaagacagagaaaatagcaa**
gatgacttagttttttttttttattctatagtctatttacgtgcagatgttgtacttgttggagaaaagacgattt
tgaatt**gtaagaaggggagagatggaatgatgaaaaatgatgctgacagtccgttgcatagtggttcttgctaca**
ggcgtggtctgtttgtttacatcgcttgtgggttacactggaatcatgttgaataacagagctattttaacctttt
tataatttattaatgtggccctgttttggtatgattgccgctattggttacacagcttatcgaaagaataagtgg
aatattgaaggaaagctatcttatcagtggcattatacattgaattcggatggcagagcacgtattcaagccaac
gtaagtcaacgggtcactttatgccactatactcatcctatgcctatagcttcactgctgtggttaccggtcgtt
ttcagattatcacgaaagatccaataaatgcttccctcgtaccttattacctggttgtaaattcaagtatcagac
attcacaaaggaagcgttgaacatcacttggattgtggctttctcaatgatacctgttcatctctttgtcttgtt
ctctggattattatgttcgaatcacatcaatcgtaaattcggcaaaggacttccacccaagatttatcgtttgga
ttatcaaggtattgttgctggtacacctactggaagttcccttaatttgcacacggatggtttacaacaaagaca
tgttcaataa

>RO3G_08988_CDS_correction of RO3G_08988 using EST
atgaacccaacactcacagcagcagccaggggacatccttatgtgtctcaagccagttcatccgccagcctacag
gacatcaggcctaatccaaggccttatgattcctatgaatcatttcctatcaatggcttacaagagcctgtcttg
ccttggatgaaatcagaatcgaggcagtcattaggatctcgtagttcgcttgatacaaccagtcttcatatcatg
aaaggtcaattccccgatccttcagctaaaaaaaaacaaaggtggtcaaagcataaatggtggttactcttatca
aatactttgttattttgttatggattagttatattattattagcactttaactttttttaaattctatttacgt
gcagatgttgtacttgttggagaaaagacgattttgaattgcgtggtctgtttgtttacatcgcttgtgggttac
actggaatcatgttgaataacagagctattttaacctttttataatttattaatgtggccctgttttggtatgatt
gccgctattggttacacagcttatcgaaagaataagtggaatattgaaggaaagctatcttatcagtggcattat
acattgaattcggatggcagagcacgtattcaagccaaccttcactgctgtggttaccggtcgttttcagattat
cacgaaagatccaataaatgcttccctcgtaccttattacctggttgtaaattcaagtatcagacattcacaaag
gaagcgttgaacatcacttggattgtggctttctcaatgatacctgttcatctctttgtcttgttctctggatta
ttatgttcgaatcacatcaatcgtaaattcggcaaaggacttccacccaagatttatcgtttggattatcaaggt
attgttgctggtacacctactggaagttcccttaatttgcacacggatggtttacaacaaagacatgttcaataa

>RO3G_08988_ correction of RO3G_08988 using EST
mnptltaaarghpyvsqasssaslqdirpnprpydsyesfpinglqepvlpwmksesrqslgsrssldttslhim
kgqfpdpsakkkqrwskhkwwlllsntllfcyglvilllalltffkfylradvvlvgektilncvvclftslvgy
tgimlnnrailtfynllmwpcfgmiaaigytayrknkwniegklsyqwhytlnsdgrariqanlhccgyrsfsdy
hersnkcfprtllpgckfkyqtftkealnitwivafsmipvhlfvlfsgllcsnhinrkfgkglppkiyrldyqg
ivagtptgsslnlhtdglqqrhvq

>Bc_Tsp3
MDVGKKLIPWAMPLLLVILTAVAGYAYSQIRALSLPISQALALFTVVLPLVTGISTQGAVGLIQRANKKEQNQLT
LPLIAVIGFQLVYETIVATLALTYMIPPKSLHCGLEDAWHSLFSSKDDRKISAIQDALNCCGLRSLADMASPIKN
PKGPGSCAALTNRSQSCLGPWRQAEQINAGLLLLVAIVIFTIKVISITNLLTTSFWRRSHWSRSIHGVTSGDTEA
PEEDNRAETRRLIEEGDDDEESYRDEPTQGRLSGLNGSGHGQGPRVEPSRLSQNY

>Ss_Tsp3_sclerotinia correction of SS1G_04021.1 using EST
atgcacgttgttaagaagttactaccatgggcaacgccactt**gtgagcactatcaagattacttggctatccaaa**
tgtacccggctaacaatatctagctgttgctcattttgactgttgtagctat**gtaggaagctccgaagcacattt**
tgatgaccaccatagactaatacctgctagctattcctactctcaaatccgcctcctttcacttcccatctcaca
agccctcgcactattcacgattgttttgccgcttgttactggtatttctacacaaggtgccattggtttgatcca
gcgagcgaacaagaaggaacaaaatcaacttacgctgccacttattgcggttattggatttcagttgatctacga
gactgttgtcgctactctggccctgacatatatgataccacccaattcattacattgtggcttggaagataaatg
gcagaagctcttcagcacgaagaatgaaggaaatattaaagcgattcaagacacgttgaattgctgtggacttca
ttctgtgtttgacaaggcatggccattcaggaaggatgtccatggacctgggaattgtgtggatttgacaaatcg
ctctca**gtgagtccttttctgacctcacgatgacatatccgaaaatccaagctaatacttagctag**gagttgttt
tggtagctggagacaagcggaacaggtcaatgccggcctgtttctcatcgtggctatagtcattttcatcattaa
g**gtacgttgttttattatcgtattaaaatgccatctacctgcaatacatgtcgcacattatagatgcgtctatt**
agtatcaaagttttatgttgtgagtggtgagataggcagatggttgctcaaggctctagaaagcctaagaaaatc
gcatttacacttgtgcacatattaaatggtttcattggtagcagagaacactccgtcctgcggtggatctaactg
tgtcattcatcttctcacatcttatccccaggtggtttccatcataaatctgctcaccaactccttctggaggcg
ctcatactggtcccagccgatccacagcataatgggtggtgatacagaggcgccacaggcagacgatagggcaga
gaccagacggcttattgaagaacgtgatgacgaagaagagtcttaccaagatgagcccactgaagatcgcctctc
accgttcaatggttctggacaaggaccaagagtcgagccttctcgcttgtctcaaaactattaa

>Ss_Tsp3_sclerotinia CDS correction of SS1G_04021.1 using EST
atgcacgttgttaagaagttactaccatgggcaacgccacttctgttgctcattttgactgttgtagctatctat
tcctactctcaaatccgcctcctttcacttcccatctcacaagccctcgcactattcacgattgttttgccgctt
gttactggtatttctacacaaggtgccattggtttgatccagcgagcgaacaagaaggaacaaaatcaacttacg
ctgccacttattgcggttattggatttcagttgatctacgagactgttgtcgctactctggccctgacatatatg
ataccacccaattcattacattgtggcttggaagataaatggcagaagctcttcagcacgaagaatgaaggaaat
attaaagcgattcaagacacgttgaattgctgtggacttcattctgtgtttgacaaggcatggccattcaggaag
gatgtccatggacctgggaattgtgtggatttgacaaatcgctctcagagttgttttggtagctggagacaagcg
gaacaggtcaatgccggcctgtttctcatcgtggctatagtcattttcatcattaaggtggtttccatcataaat
ctgctcaccaactccttctggaggcgctcatactggtcccagccgatccacagcataatgggtggtgatacagag
gcgccacaggcagacgatagggcagagaccagacggcttattgaagaacgtgatgacgaagaagagtcttaccaa
gatgagcccactgaagatcgcctctcaccgttcaatggttctggacaaggaccaagagtcgagccttctcgcttg
tctcaaaactattaa

>Sc_TSP3_sclerotinia_sclerotiorium_ correction of SS1G_04021.1 using EST
mhvvkkllpwatplllliltvvaiysysqirllslpisqalalftivlplvtgistqgaigliqrankkeqnqlt
lpliavigfqliyetvvatlaltymippnslhcgledkwqklfstknegnikaiqdtlnccglhsvfdkawpfrk
dvhgpgncvdltnrsqscfgswrqaeqvnaglflivaivifiikvvsiinlltnsfwrrsywsqpihsimggdte
apqaddraetrrlieerddeeesyqdeptedrlspfngsgqgprvepsrlsqny

>Mg_Tsp3_ annotated gene using cDNA
atgccgggaattcttccattggtggttatgggattg**gttcgcaacgttcctcctaccccaagctgcgccaaaata**
atccagtgctaaccgatcgccaccgacatggtagctctttctgggtgttgtgggcgctgccat**gtgagtactctc**
tttgatgccacctatattctcatccgctcttgctaacgactcctgatgttgattctcaaccacagctacgaaata
gtccacgcaacccagttgtccctcccgacttacccgctcaccatccccgttcttctactgccactcctcgccctc
gcaaacaccctattcgccgtccggagcaccgccctccgcctccgcgcccgcggcgacctcgccaccgccaagccc
ttcccaccgcaccccacgacgctcgtcacaatattgcagatcatccaggccatcattgccgccgtgctcgcgacc
ctcctggcagcgaccccggccgtcgactcctgcctcctcgagcagaggtggcagcatatgtacagcgcccacgac
ggtgacggaatccgacgtatccaggatgccctcggctgctgtggcttcaactcgccccgcgacagagcttggccg
ttcccgcatggaaggggaggtcagcagactaggtgtgaggaggcgtggggaaggcatgggagctgccgggcgccg
tggacggatgcgatgagggttggcgtgggtgttgagttgggggttgtggttgttgtggagattgtgcag**gtaaga**
aaccttgttgcctttggctcgcaggagccagtgaccaattgacgatgcgatagattactgatgctttgtcgaata
cgctaccgtttaggtcctggtcttactcgttcttaccggcttcccttacgtcagacgcggtctctggtatatcat
gaaccatcagacgaaccgcagagaatatccagtccacaacgcatggcgcgagcgctgtacccacgatcgtgagcg
caatggagacctatacagagacagacctctgcttgccgccgagggctcggatgcggaatcgtgcgatgaggatga
agatgctactaatgagcagccatgtggtcattgcggacggaaaagtgacggagagcagacgcaggagtctgccaa
caacttgctggctctagactcctcgattgttccaaacgtgtggagaggggacacttga

>Mg_Tsp3_cDNA
atgccgggaattcttccattggtggttatgggattgctctttctgggtgttgtgggcgctgccatctacgaaata
gtccacgcaacccagttgtccctcccgacttacccgctcaccatccccgttcttctactgccactcctcgccctc
gcaaacaccctattcgccgtccggagcaccgccctccgcctccgcgcccgcggcgacctcgccaccgccaagccc
ttcccaccgcaccccacgacgctcgtcacaatattgcagatcatccaggccatcattgccgccgtgctcgcgacc
ctcctggcagcgaccccggccgtcgactcctgcctcctcgagcagaggtggcagcatatgtacagcgcccacgac
ggtgacggaatccgacgtatccaggatgccctcggctgctgtggcttcaactcgccccgcgacagagcttggccg
ttcccgcatggaaggggaggtcagcagactaggtgtgaggaggcgtggggaaggcatgggagctgccgggcgccg
tggacggatgcgatgagggttggcgtgggtgttgagttgggggttgtggttgttgtggagattgtgcaggtcctg
gtcttactcgttcttaccggcttcccttacgtcagacgcggtctctggtatatcatgaaccatcagacgaaccgc
agagaatatccagtccacaacgcatggcgcgagcgctgtacccacgatcgtgagcgcaatggagacctatacaga
gacagacctctgcttgccgccgagggctcggatgcggaatcgtgcgatgaggatgaagatgctactaatgagcag
ccatgtggtcattgcggacggaaaagtgacggagagcagacgcaggagtctgccaacaacttgctggctctagac
tcctcgattgttccaaacgtgtggagaggggacacttga

>Mg_Tsp3
mpgilplvvmgllflgvvgaaiyeivhatqlslptypltipvlllpllalantlfavrstalrlrargdlatakp
fpphpttlvtilqiiqaiiaavlatllaatpavdsclleqrwqhmysahdgdgirriqdalgccgfnsprdrawp
fphgrggqqtrceeawgrhgscrapwtdamrvgvgvelgvvvvveivqvlvllvltgfpyvrrglwyimnhqtnr
reypvhnawrercthdrerngdlyrdrpllaaegsdaescdededatneqpcghcgrksdgeqtqesannllald
ssivpnvwrgdt

>Pa_Tsp3 Podospora_manually annotated gene
Atggtcctcctcctcgccctctacgtcctg**gtatgccaccaccctgacctttaatcaccccaatgctaaccctttt**
tcccagctaatcatcaccctaaccggcctcgccat**gtctgtccccccgtttccccttttcccctcgccatcactaa**
cctttatcccagctaccaacaccacacaagcaccaccctctccctcccccctctcccccaccctcacaatcctcac
catcctcctcccccctcctctcccttctcaccacctccctaaccttcttctcctcctcccaccccccgcaaacccct
cccccccctttttatccaacgctctccaactcctcctcaccatcatcttgtccaccctgttcggcgccaccctcac
atccccctatctcccctgcgccctccaaacaacctggcgatccctctggacatcccactccgccacccccatccg
cacgattcaagactccctctcctgctgcggcttcaaatcaaccaaggacatggcctggcctttttccctcgagcgg
caacaacggcgatgtttccagctgcgagaagcagttcaaccggcacacaccctgcgctggaccgtgggaagacgc
cctgaacaaaacgaccggggtggagctgggtgttgttgtggtagcgggggttgttcagcttctttcactg**gtggt**
gtttaaaacacagcggaggggggggcaagggaaaaaggtgtggttggggagggtggtggagatttttactggaca
ggacgatgaagggcaggggagacggccgcttttgacggggggggaggggtcacagtgaggaagggaggtatattga
cgggggagtggaggaggaggagcagggtggtcacgaaaatggcaatggctatggagggacctcaagctcagccca
gacgcaaacacaaccagacgacaataacagccgtggtggaccaagggtcgaagtctcccatcatgacccctgggc
cggggcggagagggtctaa

>Pa_TSP3_podospora_anserina_CDS manual annotation
atggtcctcctcctcgccctctacgtcctgctaatcatcaccctaaccggcctcgccatctaccaacaccacaca
agcaccaccctctccctcccccctctcccccaccctcacaatcctcaccatcctcctcccccctcctctcccttctc
accacctccctaaccttcttctcctcctcccaccccccgcaaacccctcccccccctttttatccaacgctctccaa
ctcctcctcaccatcatcttgtccaccctgttcggcgccaccctcacatccccctatctcccctgcgccctccaa
acaacctggcgatccctctggacatcccactccgccacccccatccgcacgattcaagactccctctcctgctgc
ggcttcaaatcaaccaaggacatggcctggcctttttccctcgagcggcaacaacggcgatgtttccagctgcgag
aagcagttcaaccggcacacaccctgcgctggaccgtgggaagacgccctgaacaaaacgaccggggtggagctg
ggtgttgttgtggtagcgggggttgttcagcttctttcactggtgtggttggggagggtggtggagatttttact
ggacaggacgatgaagggcaggggagacggccgcttttgacggggggggaggggtcacagtgaggaagggaggtat
attgacgggggagtggaggaggaggagcagggtggtcacgaaaatggcaatggctatggagggacctcaagctca
gcccagacgcaaacacaaccagacgacaataacagccgtggtggaccaagggtcgaagtctcccatcatgacccc
tgggccggggcggagagggtctaa

>Pa_Tsp3_ manually annotated protein
mvlllalyvlliitltglaiyqhhtsttlslplsptltiltillpllsllttsltffssshprkplppllsnalq
llltiilstlfgatltspylpcalqttwrslwtshsatpirtiqdslsccgfkstkdmawpfpssgnngdvssce
kqfnrhtpcagpwedalnkttgvelgvvvvagvvqllslvwlgrvveiftgqddegqgrrplltggrghseegry
idggveeeeqgghengngyggtsssaqtqtqpddnnsrggprvevshhdpwagaerv

>An_Tsp3 annotated gene defined using EST
atggcagttatctacggattgcccacggtggcttccacagtagcagcagcg**gtatgagcaagtccactgttcttg**
aacgccaactaacaagtcatcgtctccaatagtgtgcgataattgctatcattcttggagc**gtaagtcaccttct**
cacacttgatacagcggtgtggcgtcgagaaccaagcttaatcatctcttcaggctgtcctggtctcgtacagct
tcactttaccttccccttcctgaatgggttccagccatcgccaccatcttccctcctcttacagcactggctctg
taccttgccagtcgactcgcccagcctgcagatgaccgccagtcgtctagcccttggcgaagactccttcccgtc
atgaaccacctccaatctatcataaccacaatcattgccaccgtggccctcgcctacttgtacccagagagcatc
acaacctgtcgactggaacaggaatggcagtcatacttccgcgcgaaagacgctcaacctattcgcgctattcag
gacgaatttcgctgttgtggattccggagtatccatgaccgagcgtggccgttcaaggacaaaaccaccggagat
gatgcatgtgaggtgcagtttaattacgggacaagctgtctggtcccgtggagacagcagcagcagagtgcttca
tggatggtttttgtagcagctgttttgactttgctcatgaaggttcgt**gtatgcgcttcgttactgttaatgatt**
tactaaggaaaactacaggttgcgttataccaggccatgcgtcagagaccgagctggatgacaatgcagtttgga
agacaggcacgcagccagcagtatattacgcccgctgcgctggaggatgaagacgccaatgacgacgaggagggg
gggcggcggggagcgttcctgcctcgtgcgggtccaaggcttgacaatgagtggaatgatcggtag

>An_Tsp3_cDNA defined using EST
atggcagttatctacggattgcccacggtggcttccacagtagcagcagcgtgtgcgataattgctatcattctt
ggagcgctgtcctggtctcgtacagcttcactttaccttccccttcctgaatgggttccagccatcgccaccatc
ttccctcctcttacagcactggctctgtaccttgccagtcgactcgcccagcctgcagatgaccgccagtcgtct
agcccttggcgaagactccttcccgtcatgaaccacctccaatctatcataaccacaatcattgccaccgtggcc
ctcgcctacttgtacccagagagcatcacaacctgtcgactggaacaggaatggcagtcatacttccgcgcgaaa
gacgctcaacctattcgcgctattcaggacgaatttcgctgttgtggattccggagtatccatgaccgagcgtgg
ccgttcaaggacaaaaccaccggagatgatgcatgtgaggtgcagtttaattacgggacaagctgtctggtcccg
tggagacagcagcagcagagtgcttcatggatggtttttgtagcagctgttttgactttgctcatgaaggttcgt

gttgcgttataccaggccatgcgtcagagaccgagctggatgacaatgcagtttggaagacaggcacgcagccag
cagtatattacgcccgctgcgctggaggatgaagacgccaatgacgacgaggagggggggcggcggggagcgttc
ctgcctcgtgcgggtccaaggcttgacaatgagtggaatgatcggtag

>An_Tsp3 defined using EST
maviyglptvastvaaacaiiaiilgalswsrtaslylplpewvpaiatifppltalalylasrlaqpaddrqss
spwrrllpvmnhlqsiittiiatvalaylypesittcrleqewqsyfrakdaqpiraiqdefrccgfrsihdraw
pfkdkttgddacevqfnygtsclvpwrqqqsaswmvfvaavltllmkvrvalyqamrqrpswmtmqfgrqarsq
qyitpaalededanddeeggrrgaflpragprldnewndr*

>Tr_Tsp3
malglglvfllgsavlfgvaavvhfhsahlslpinpaitiltvllpiisllnsyiyptllhsahhssnpfhrlsp
tilqtlqglltvlatllfedvlpsttvecllldnqwlrmfrakdgesirliqdtlnccglnsvkdrafpwpkpdq
kysicaemfgrsqacrgpwraalrssagadfgvvvavgllqilsllmtregtnwwnawrsinwgrrqrirhsesr
plledvtdadevverqdesphprgyqslpandqdnrprvepstihhernawnde

>Gz_Tsp3
MIHPTVLYLTLSVALLVIAVIVHVRSSNLSLAISPAVSIITVILPIVGFLNTACYPSFRRTTKSSPSRVAQLGPL
VVQVLQALITTILATLLFERALPSGVTNCMMENQWMSMFRAHDAGGIRRIQDAFDCCGFNSVRDRAYPFPGTAPS
TCAATYGRRTACREPWQGALQTNAMADFAVVVGVGLIQIMGLFVVPESGNWRNAWGVNRSGERNQQSESRRPLLI
DRERDVVEEEETEPEQPEGESQGYGSLNANVSGSRVVPSSVAERNNWADE

>Nc_Tsp3_genomic_hypothetical_manually annotated gene
atgattaccctaggcttgatctacaccttg**gtctgccaccacattcagccttcatacaattgacgttgttgccga**
caagattgactgacacctctcaacagatcatcctcaccctcctggccttcgccatatatgaacatatccacgccc
aatccctttctctccccatttccccaggtctaaccatcttgaccatcctcctccccatcctctccgctctcaaca
ccgcctacctctaccgcgtcaccactaccacga**gtcccagccaaccaaacccaccaaagttcataactgcccact**
cctcctcaaccataaccccaaccccctccaacccctctcaacaactcacccacctccgcccagctcacgccccgc
gccgaccacaacaatcttctactagcccaggacccctccgctgcctcctccccaccctctcccaaaccctgcaaa
caacccaacttgtcctctctctcatcctttttgagtgtagccacctcctccctgacttcctccccctttttcct**ctt**
tccccaactccggcccggggtcaatcgaacactgcgccctcgaacaaacctggtcgcacttctttcggtcgcacg
acgccgacaccatccgcaagatccaggatatcctgggatgctgtgggttccgaagtccgaagcacatgagttggc
cttttccaagcgcaggaaagggtacggagcaatgtggacagatgtggcctgagagaagggaggggtgtgccggga
agtgggaaggggagtttagggggggtgatgggtggggagattggagttgtggcgggggtgttggtggtgcagattt
tggggtggggggtggggag**gtggatggcgacgagaagagcgaggacgggggcaacgaggaggagaggaggacagg**
atgggagggctacgatgggcggaagaggaagggaggggagtgggatggtaaatggtgagggacgtgatggacagg
gaggagatggaaataatagctggtggaataaactcttacagagctttggggtgccacatggggatgaggaggatg
gctatcggggattggaagatggaggagctcaacggaggccattgcttgatgcgggacagaatggagagggccatg
ttgggattgtggaggtagatgatgatgaagatggagagggaagggacgttgaaaggggagggagtgatgaggaga
gtaggtcgggtagtgagagaaggggcgggaacggatatggaagtgctacttcgagggtgcagccgagtggcattc
atgttgctgatccatgggccgaagggaattga

>Nc_Tsp3_CDS_manual_annotation
atgattaccctaggcttgatctacaccttgatcatcctcaccctcctggccttcgccatatatgaacatatccac
gcccaatccctttctctccccatttccccaggtctaaccatcttgaccatcctcctccccatcctctccgctctc
aacaccgcctacctctaccgcgtcaccactaccacgagacccctccgctgcctcctccccaccctctcccaaacc
ctgcaaacaacccaacttgtcctctctctcatcctttttgagtgtagccacctcctccctgacttcctcccccttt
tcctactgcgccctcgaacaaacctggtcgcacttctttcggtcgcacgacgccgacaccatccgcaagatccag
gatatcctgggatgctgtgggttccgaagtccgaagcacatgagttggccttttccaagcgcaggaaagggtacg
gagcaatgtggacagatgtggcctgagagaagggaggggtgtgccgggaagtgggaaggggagtttagggggggtg
atgggtggggagattggagttgtggcgggggtgttggtggtgcagattttggggtggggggtggggagctggtgg
aataaactcttacagagctttggggtgccacatggggatgaggaggatggctatcggggattggaagatggagga
gctcaacggaggccattgcttgatgcgggacagaatggagagggccatgttgggattgtggaggtagatgatgat
gaagatggagagggaagggacgttgaaaggggagggagtgatgaggagagtaggtcgggtagtgagagaaggggc
gggaacggatatggaagtgctacttcgagggtgcagccgagtggcattcatgttgctgatccatgggccgaaggg
aattga

>Nc_Tsp3_hypothetical_ manually annotated protein
mitlgliytliiltllafaiyehihaqslslpispgltiltillpilsalntaylyrvttttrplrcllptlsqt
lqttqlvlslillsvatssltsspfsycaleqtwshffrshdadtirkiqdilgccgfrspkhmswpfpsagkgt

eqcgqmwperregcagkwegefrgvmggeigvvagvlvvqilgwgvgswwnkllqsfgvphgdeedgyrgledgg
aqrrplldagqngeghvgivevdddedgegrdverggsdeesrsgserrggngygsatsrvqpsgihvadpwaeg
n

>Ur_Tsp3
MPFKIDTTTPLYIVLVVGGAISLILGALSWARTAALLLPLPTWVPATATLISPITVLTLIATRVFSKQSDHETPR
NCWWSTISGILNQIQTIISTIVATVALAYIFPDSILSCNLDQQWQAFFQSKNSHAIRSIQDEFRCCGLRSLHDRA
WPFKDRNHGDNACELQLGYQRSCFAPWREHQQSTSWMVFAAAVFVFAAKIAYIRLFSHRMSWMSTQSAIRRPDYQ
QIIHTAVQDEGDNENGVHGEAQRTFLPESITEYRNDWEVD
