# Supplementary material for: Fungi have three tetraspanin families with distinct functions
Source: BMC Genomics. 2008 Feb 3;9:63. doi: 10.1186/1471-2164-9-63 (PMC2278132; doi:10.1186/1471-2164-9-63)
Supplement: Additional File 7 — Primers used for this study. Primers used for genes tested by quantitative RT-PCR and for targeted gene replacement of TSP3 and TPL1 ORFs in M. grisea. [file 1471-2164-9-63-S7.PDF]

Additional file 7

Name		Sequence 5'-3'	Purpose
ILV5 (MGG_01808.5)	Forward	CCAGCTCTACGACTCGGTCAA	qPCR
	Reverse	AGTCGGGCTGGCTGTTGTAGT	qPCR
PLS1	Forward	CAGCAGCTTCAGCCTTGATG	qPCR
	Reverse	AAGACGTTGGCGAAGCTAGTG	qPCR
TSP3	Forward	TCTTACCGGCTTCCCTTACGT	qPCR
	Reverse	TCTCTGCGGTTCGTCTGATG	qPCR
TPL1 (MGG_08113.5)	Forward	GTCCTGAGGAGGGCAACGA	qPCR
	Reverse	GGAAAGCAAAATCTGCCTGAGTA	qPCR
KO1$^{\text{MGG_07922.5}}$	Forward	CAACGAGGAGAAGAACTACGGCG	*TSP3* deletion
KO2$^{\text{MGG_07922.5}}$	Reverse	CAC*GGCCTGAGTGGCC* GCCGATTCCAGTTCCTTGTCAT	*TSP3* deletion
KO3$^{\text{MGG_07922.5}}$	Forward	GTG*GGCCATCTAGGCC* CCGTGTGGCTTCTTCTGGTGCT	*TSP3* deletion
KO4$^{\text{MGG_07922.5}}$	Reverse	CCTCCTTGGTCTTGCCCTCCC	*TSP3* deletion
KO5$^{\text{MGG_07922.5}}$	Forward	GCTACGGCTACAACGAGGACACC	*TSP3* deletion
KO6$^{\text{MGG_07922.5}}$	Reverse	GTGATGCTGCCGACGCTGC	*TSP3* deletion
KO1$^{\text{MGG_08113.5}}$	Forward	AGCTTGCTTCGTCTACTGTAGACC	*TPL1* deletion
KO2$^{\text{MGG_08113.5}}$	Reverse	CAC*GGCCTGAGTGGCC* AATGCCACCAGCTAGACGTTG	*TPL1* deletion
KO3$^{\text{MGG_08113.5}}$	Forward	GTG*GGCCATCTAGGCC* ACGAGTACCACGGGACAAGGC	*TPL1* deletion
KO4$^{\text{MGG_08113.5}}$	Reverse	GTATTTGCAGGAAACTTGATGACCAC	*TPL1* deletion
KO5$^{\text{MGG_08113.5}}$	Forward	GCGGATACCTCAGGAACTCTCACC	*TPL1* deletion
KO6$^{\text{MGG_08113.5}}$	Reverse	ATCTCCGCATGGACAGGTGGG	*TPL1* deletion
